# No Benefit of Hemostatic Drugs on Acute Upper Gastrointestinal Bleeding in Cirrhosis

**DOI:** 10.1155/2020/4097170

**Published:** 2020-06-26

**Authors:** Yang An, Zhaohui Bai, Xiangbo Xu, Xiaozhong Guo, Fernando Gomes Romeiro, Cyriac Abby Philips, Yingying Li, Yanyan Wu, Xingshun Qi

**Affiliations:** ^1^Department of Gastroenterology, General Hospital of Northern Theater Command (formerly General Hospital of Shenyang Military Area), Shenyang 110840, China; ^2^Postgraduate College, Shenyang Pharmaceutical University, Shenyang 110016, China; ^3^Department of Internal Medicine, Botucatu Medical School, UNESP-Univ Estadual Paulista. Av. Prof. Mário Rubens Guimarães Montenegro, s/n Distrito de Rubião Jr, Botucatu, Brazil; ^4^The Liver Unit and Monarch Liver Lab, Cochin Gastroenterology Group, Ernakulam Medical Center, Kochi, 682028 Kerala, India; ^5^Department of Gastroenterology, The First People's Hospital of Huainan, Huainan 232007, China; ^6^Postgraduate College, Jinzhou Medical University, Jinzhou 121001, China

## Abstract

**Background and Aims:**

Acute upper gastrointestinal bleeding (AUGIB) is one of the most life-threatening emergency conditions. Hemostatic drugs are often prescribed to control AUGIB in clinical practice but have not been recommended by major guidelines and consensus. The aim of this study was to investigate the therapeutic effect of hemostatic drugs on AUGIB in cirrhosis.

**Methods:**

All cirrhotic patients with AUGIB who were admitted to our hospital from January 2010 to June 2014 were retrospectively included. Patients were divided into hemostatic drugs and no hemostatic drug groups. A 1 : 1 propensity score matching (PSM) analysis was performed by adjusting age, gender, etiology of liver disease, Child-Pugh score, MELD score, hematemesis, red blood cell transfusion, vasoactive drugs, antibiotics, proton pump inhibitors, and endoscopic variceal therapy. Primary outcomes included 5-day rebleeding and in-hospital mortality.

**Results:**

Overall, 982 cirrhotic patients with AUGIB were included (870 in hemostatic drugs group and 112 in no hemostatic drug group). In overall analyses, hemostatic drugs group had a significantly higher 5-day rebleeding rate (18.10% versus 5.40%, *P* = 0.001) than no hemostatic drug group; in-hospital mortality was not significantly different between them (7.10% versus 4.50%, *P* = 0.293). In PSM analyses, 172 patients were included (86 patients in each group). Hemostatic drugs group still had a significantly higher 5-day rebleeding rate (15.10% versus 5.80%, *P* = 0.046); in-hospital mortality remained not significantly different (7.00% versus 3.50%, *P* = 0.304) between them. Statistical results remained in PSM analyses according to the type of hemostatic drugs.

**Conclusions:**

The use of hemostatic drugs did not improve the in-hospital outcomes of cirrhotic patients with AUGIB.

## 1. Introduction

Acute upper gastrointestinal bleeding (AUGIB) is a life-threatening and frequent complication in cirrhosis with its mortality approaching 5-20% [1–4]. About 70% of AUGIB episodes in cirrhosis are due to esophageal variceal rupture secondary to portal hypertension [5]. The primary goals of therapy of AUGIB in liver cirrhosis are initial control of bleeding and prevention of early rebleeding [1, 5, 6]. According to the current guidelines, the mainstay pharmacological management of AUGIB should be the use of vasoactive drugs (terlipressin and somatostatin or its analogues), which can reduce portal blood flow and portal pressure [6–8].

Traditionally, it has been considered that variceal rupture bleeding is potentially more dangerous in cirrhosis due to the underlying coagulation abnormalities [9, 10]. In clinical practice, though not recommended, treating physicians arbitrarily prescribe hemostatic drugs, which act on vasculature or coagulation cascade, as adjuvants for control of bleeding [11]. However, the therapeutic effect of hemostatic drugs on AUGIB remains uncertain. The results of a recent meta-analysis showed that antifibrinolytic agents were deleterious in patients with acute or chronic liver disease and AUGIB [12]. Herein, we conducted a retrospective study to investigate the effect of hemostatic drugs on AUGIB in patients with liver cirrhosis.

## 2. Methods

The study was approved by the Medical Ethical Committee of the General Hospital of Northern Theater Command with an approval number [number K (2019)32] and was performed according to the Declaration of Helsinki.

### 2.1. Study Design

In this retrospective study, a total of 1026 cirrhotic patients with AUGIB who were consecutively admitted to the General Hospital of Northern Theater Command from January 2010 to June 2014 were screened. The inclusion criteria were as follows: (1) a diagnosis of liver cirrhosis, and (2) a diagnosis of AUGIB presenting with hematemesis and/or melena at admission. The exclusion criteria were as follows: (1) no episodes of gastrointestinal bleeding within 5 days before admission, and (2) only positive occult blood test. Age, sex, source of gastrointestinal bleeding, cause of liver disease, and malignancy were not limited. Repeated admission was not excluded. Finally, 982 patients were included in our study.

The following data was extracted from our retrospective database: demographic data (i.e., age and gender), etiology of liver disease, presence of hematemesis and/or melena at admission, and laboratory tests (i.e., red blood cell, hemoglobin, white blood cell, platelet count, total bilirubin, direct bilirubin, albumin, alanine aminotransferase, aspartate aminotransferase, alkaline phosphatase, gamma-glutamyl transpeptidase, blood urea nitrogen, creatinine, potassium, sodium, prothrombin time, activated partial thromboplastin time, and international normalized ratio [INR]). The Child-Pugh score and model for end-stage liver disease (MELD) score were calculated. The use of red blood cell transfusion and antibiotics as well as the use of vasoactive drugs (i.e., somatostatin and/or octreotide) and proton pump inhibitors (PPIs) were recorded. The grade of esophageal varices was evaluated [13]. The use of endoscopic variceal therapy, Sengstaken-Blakemore tube, and splenectomy with or without devascularization were also recorded.

Hemostatic drugs employed in our study included drugs acting on vascular wall or platelet (i.e., norepinephrine, carbazochrome sodium sulfonate, and Yunnan Baiyao), antifibrinolytic drugs (i.e., ethylenediamine diacetoacetic), thrombin (i.e., lyophilizing thrombin powder), hemocoagulase (i.e., snake venom hemocoagulase), and procoagulant drugs (i.e., vitamin K). Modes of administration included intravenous, oral, and topical administration. The mechanisms and indications of various hemostatic drugs are shown in Supplementary Table 1.

According to the use of these hemostatic drugs during hospitalization, we divided the patients into hemostatic drugs and no hemostatic drug groups. The major outcomes included a 5-day rebleeding rate and in-hospital mortality. Five-day rebleeding was defined as the recurrence of hematemesis and fresh melena within 5 days after the initial bleeding episode was completely controlled [3].

### 2.2. Statistical Analyses

Continuous variables were expressed as mean ± standard deviation and median (range). Categorical variables were expressed as frequency (percentage). Nonparametric Mann-Whitney *U* test was used for continuous variables, and chi-square test was used for categorical variables to compare the differences between hemostatic drugs and no hemostatic drug groups. A 1 : 1 propensity score matching (PSM) analysis was used. Matching factors included age, gender, etiology of liver diseases, which mainly include hepatitis B, hepatitis C, alcohol abuse, drug abuse, and autoimmunity, Child-Pugh score, MELD score, hematemesis, red blood cell transfusion, vasoactive drugs, antibiotics, PPIs, and endoscopic variceal therapy. After exclusion of patients with malignancy and those who underwent surgery, subgroup analyses were conducted in patients with Child-Pugh class B and C, MELD score > 15 [14], and use of endoscopic variceal therapy and antibiotics. All statistical analyses were performed with IBM SPSS software version 20.0 (IBM Corp, Armonk, NY, USA) and Stata/SE 12.0 (Stata Corp, College Station, TX, USA) software. A histogram demonstrating the frequency of various hemostatic drugs used during the study period was drawn by the Excel version 10.0 (Microsoft Corp, Redmond Washington, USA).

## 3. Results

### 3.1. Overall Analyses

A total of 982 patients with cirrhosis and AUGIB were included in our study. Baseline characteristics of patients at admission were shown in Table 1. The median age was 56.01 years (range: 6.28-95.13 years), and most patients were male (*n* = 688, 70.1%). Hepatitis B virus (*n* = 399, 40.6%) was the most common etiology of cirrhosis. One hundred and eighty-nine patients (19.2%) had malignancy, including 160 patients with liver cancer and 29 patients with extrahepatic cancer (i.e., lung cancer, breast cancer, gastric cancer, and rectal cancer). Most patients were in Child-Pugh class B (476/982, 52.80%). The median MELD score was 6.60 (range: -7.52-40.95). Endoscopy was performed in 702 patients. Detailed information regarding the grade of esophageal varices on endoscopy was clearly available in 563 patients. Hemostatic drugs group had a higher proportion of hematemesis at admission, lower levels of red blood cell, platelet count, albumin, and alkaline phosphatase, and higher levels of blood urea nitrogen, potassium, prothrombin time, and INR than no hemostatic drug group.

Blood transfusion was given in 640 (65.20%) patients, of whom 611 (62.20%) received red blood cell transfusion with a median of 4 units (range: 1.00-33.00). Somatostatin and/or octreotide were given in 892 (90.80%) patients. PPIs were given in 967 (98.50%) patients. Antibiotics were given in 468 (47.70%) patients. Endoscopic variceal therapy was performed in 574 (58.50%) patients. Sengstaken-Blakemore tube placement was given in 20 (1.90%) patients. Splenectomy with and without devascularization was performed in 9 (0.9%) patients. Hemostatic drugs group was more likely to receive blood transfusion, red blood cell transfusion, somatostatin and/or octreotide, and PPIs than no hemostatic drug group.

**Figure 1:**
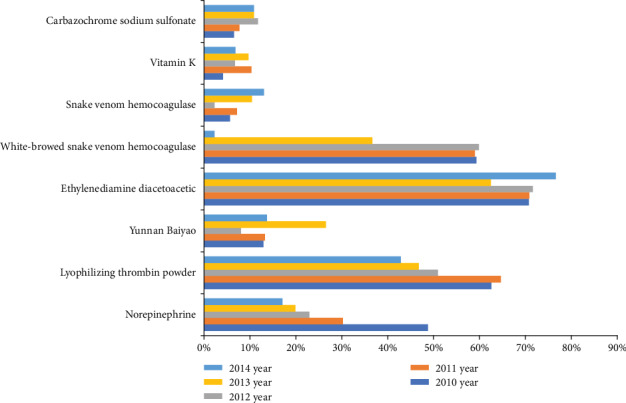
A trend in the frequency of hemostatic drugs used in our study.

Among the hemostatic drugs, ethylenediamine diacetoacetic, white-browed snake venom hemocoagulase, and lyophilizing thrombin powder were common hemostatic drugs with a high utilization rate of up to 60%-70%. By contrast, carbazochrome sodium sulfonate, vitamin K, and snake venom hemocoagulase were uncommon hemostatic drugs with a relatively low utilization rate of about 10%. There was a trend in a lower utilization rate of norepinephrine, white-browed snake venom hemocoagulase, and lyophilizing thrombin powder over time. By contrast, there was a trend in a higher utilization rate of carbazochrome sodium sulfonate and snake venom hemocoagulase over time (Figure 1).

The 5-day rebleeding rate was 16.6% (*n* = 163), and in-hospital mortality was 6.8% (*n* = 67). Hemostatic drugs group had a significantly higher 5-day rebleeding rate than no hemostatic drug group (18.10% versus 5.40%, *P* = 0.001). In-hospital mortality was not significantly different between the two groups (7.10% versus 4.50%, *P* = 0.293). The causes of death included uncontrolled bleeding (*n* = 40), uncontrolled bleeding with hepatic encephalopathy (*n* = 5), end-stage liver disease (*n* = 20), and advanced hepatocellular carcinoma (*n* = 2).

### 3.2. PSM Analyses

#### 3.2.1. PSM Analyses of Any Hemostatic Drug

A total of 172 patients were included in PSM analyses. In the hemostatic drugs group (*n* = 86), most patients (*n* = 74) started using hemostatic drugs from the day at admission until the bleeding stopped or death, and average duration of hemostatic drugs was 6.99 days (range: 1-41 days); rebleeding occurred in 13 patients during hospitalization, all of which developed after the use of hemostatic drugs. Compared with no hemostatic drug group, hemostatic drugs group had a significantly higher incidence of 5-day rebleeding (15.10% versus 5.80%, *P* = 0.046). In-hospital mortality was statistically similar between the two groups (7.00% versus 3.50%, *P* = 0.304) (Table 2).

#### 3.2.2. PSM Analyses of Ethylenediamine Diacetoacetic

A total of 160 patients were included in PSM analyses. In the ethylenediamine diacetoacetic group (*n* = 80), rebleeding occurred in 10 patients during hospitalization, all of which developed after the use of ethylenediamine diacetoacetic. There was no significant difference in the incidence of 5-day rebleeding (13.30% versus 5.30%, *P* = 0.092) or in-hospital mortality (5.30% versus 4.00%, *P* = 0.699) between the two groups (Supplementary Table 2).

#### 3.2.3. PSM Analyses of Lyophilizing Thrombin Powder

A total of 140 patients were included in PSM analyses. In the lyophilizing thrombin powder group (*n* = 70), rebleeding occurred in 10 patients during hospitalization, all of which developed after the use of lyophilizing thrombin powder. There was no significant difference in the incidence of 5-day rebleeding (14.30% versus 5.70%, *P* = 0.091) or in-hospital mortality (2.90% versus 2.90%, *P* = 1.000) between the two groups (Supplementary Table 3).

#### 3.2.4. PSM Analyses of White-Browed Snake Venom Hemocoagulase

A total of 128 patients were included in PSM analyses. In the white-browed snake venom hemocoagulase group (*n* = 64), rebleeding occurred in 10 patients during hospitalization, all of which developed after the use of white-browed snake venom hemocoagulase. There was no significant difference in the incidence of 5-day rebleeding (12.50% versus 4.70%, *P* = 0.115) or in-hospital mortality (3.10% versus 3.10%, *P* = 1.000) between the two groups (Supplementary Table 4).

#### 3.2.5. PSM Analyses of Snake Venom Hemocoagulase

A total of 62 patients were included in PSM analyses. In the snake venom hemocoagulase group (*n* = 31), rebleeding occurred in 6 patients during hospitalization, all of which developed after the use of snake venom hemocoagulase. There was no significant difference in the incidence of 5-day rebleeding (19.40% versus 9.70%, *P* = 0.279) or in-hospital mortality (9.70% versus 3.20%, *P* = 0.301) between the two groups (Supplementary Table 5).

#### 3.2.6. PSM Analyses of Yunnan Baiyao

A total of 98 patients were included in PSM analyses. In the Yunnan Baiyao group (*n* = 49), rebleeding occurred in 13 patients during hospitalization, all of which developed after the use of Yunnan Baiyao. The incidence of 5-day rebleeding was significantly higher in the Yunnan Baiyao group (26.50% versus 4.10%, *P* = 0.002). There was no significant difference in the in-hospital mortality (12.20% versus 4.10%, *P* = 0.140) between the two groups (Supplementary Table 6).

#### 3.2.7. PSM Analyses of Norepinephrine

A total of 96 patients were included in PSM analyses. In the norepinephrine group (*n* = 48), rebleeding occurred in 10 patients (6 patients were treated with norepinephrine during the endoscopic variceal therapy procedure and 4 patients were treated with norepinephrine orally) during hospitalization, all of which developed after the use of norepinephrine. The incidence of 5-day rebleeding was significantly higher in the norepinephrine group (20.80% versus 6.20%, *P* = 0.037). In-hospital mortality was significantly lower in the norepinephrine group (0.00% versus 8.30%, *P* = 0.041) (Supplementary Table 7).

#### 3.2.8. PSM Analyses of Carbazochrome Sodium Sulfonate

A total of 62 patients were included in PSM analyses. In the carbazochrome sodium sulfonate group (*n* = 31), rebleeding occurred in 3 patients during hospitalization, all of which developed after the use of carbazochrome sodium sulfonate. There was no significant difference in the incidence of 5-day rebleeding (9.70% versus 3.20%, *P* = 0.301) or in-hospital mortality (6.50% versus 0.00%, *P* = 0.151) between the two groups (Supplementary Table 8).

#### 3.2.9. PSM Analyses of Vitamin K

A total of 64 patients were included in PSM analyses. In the vitamin K group (*n* = 32), rebleeding occurred in 10 patients during hospitalization, all of which developed after the use of vitamin K. The incidence of 5-day rebleeding was significantly higher in the vitamin K group (31.20% versus 6.20%, *P* = 0.010). There was no significant difference in the in-hospital mortality (15.60% versus 3.10%, *P* = 0.086) between the two groups (Supplementary Table 9).

### 3.3. Subgroup Analyses

#### 3.3.1. Subgroup Analyses of Patients with Child-Pugh Class B and C after Excluding Patients with Malignancy and Those Undergoing Surgery

After excluding patients with malignancy and those undergoing surgery, 523 patients had Child-Pugh class B and C. Hemostatic drugs group had a significantly higher incidence of 5-day rebleeding (18.10% versus 1.90%, *P* = 0.003), but there was no significant difference in the in-hospital mortality (7.40% versus 3.80%, *P* = 0.323) between the two groups.

#### 3.3.2. Subgroup Analyses of Patients with MELD Score > 15 after Excluding Patients with Malignancy and Those Undergoing Surgery

After excluding patients with malignancy and those undergoing surgery, 79 patients had a MELD score > 15. Hemostatic drugs group had a significantly higher incidence of 5-day rebleeding (15.90% versus 4.30%, *P* = 0.003), but there was no significant difference in the in-hospital mortality (6.10% versus 3.30%, *P* = 0.278) between the two groups.

#### 3.3.3. Subgroup Analyses of Patients Having Esophageal Varices on Endoscopy after Excluding Patients with Malignancy and Those Undergoing Surgery

After excluding patients with malignancy and those undergoing surgery, 471 patients had esophageal varices on endoscopy. There was no significant difference in the incidence of 5-day rebleeding (14.90% versus 8.30%, *P* = 0.218) or in-hospital mortality (3.50% versus 2.10%, *P* = 0.596) between the two groups.

#### 3.3.4. Subgroup Analyses of Patients Receiving Endoscopic Variceal Therapy and Antibiotics after Excluding Patients with Malignancy and Those Undergoing Surgery

After excluding patients with malignancy and those undergoing surgery, 243 patients received both endoscopic variceal therapy and antibiotics. There was no significant difference in the incidence of 5-day rebleeding (18.90% versus 9.50%, *P* = 0.285) or in-hospital mortality (4.50% versus 0.00%, *P* = 0.321) between the two groups.

#### 3.3.5. Subgroup Analyses of Patients with Liver Cancer

There were 160 patients with liver cancer. There was no significant difference in the incidence of 5-day rebleeding (26.10% versus 11.10%, *P* = 0.164) or in-hospital mortality (12.00% versus 11.10%, *P* = 0.915) between the two groups.

## 4. Discussion

Hemostatic drugs have never been recommended by major practice guidelines and consensus for the management of AUGIB in liver cirrhosis [1]. This is primarily because previous studies did not find any benefit of hemostatic drugs for AUGIB [15–20], which is consistent with our findings. Notably, our overall, PSM, and subgroup analyses suggested that neither 5-day rebleeding rate nor in-hospital mortality was improved by the use of hemostatic drugs. This finding can be explained by the fact that AUGIB in cirrhosis is mainly caused by hemodynamic alterations of portal hypertension, but not coagulation disorder [21–23]. A well-known effect of vasoactive drugs is the visceral vasoconstriction, thus decreasing the portal pressure, so these drugs are the first-line choice of therapy for acute variceal bleeding [23]. By comparison, hemostatic drugs cannot act on portal pressure reduction.

**Figure 2:**
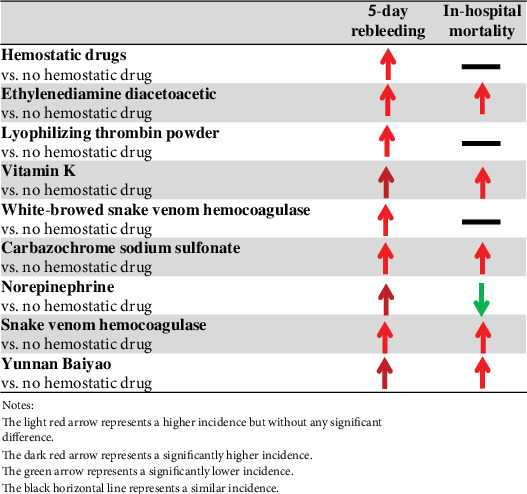
An overview of our findings.

Our study specifically analyzed the effect of different hemostatic drugs in patients with cirrhosis and AUGIB. The findings from PSM analyses performed according to the type of hemostatic drugs were similar to those from overall analysis (Figure 2).

Tranexamic acid is one of the most widely employed antifibrinolytic drugs [24]. A meta-analysis showed that the use of tranexamic acid might not reduce the mortality of AUGIB [15]. Tranexamic acid was administered to few patients in our study, but the majority of our patients received ethylenediamine diacetoacetic which has the same mechanism as tranexamic acid. Therefore, the findings of the previous meta-analysis might be comparable to our finding that ethylenediamine diacetoacetic did not improve the in-hospital outcome of cirrhosis with AUGIB.

The *ε*-aminocaproic acid is another antifibrinolytic drug. Gunawa and Runyon reported a potential benefit of *ε*-aminocaproic acid for hyperfibrinolysis, defined as abnormal euglobulin lysis time < 120 min, in liver cirrhosis [16]. Among the 37 cirrhotic patients with hyperfibrinolysis who developed bleeding episodes and received *ε*-aminocaproic acid, the hemostatic successful rate was 92% (34/37). However, a control group without *ε*-aminocaproic acid was lacking and the findings might be inconclusive. By comparison, euglobulin lysis time was not regularly measured in our study, and the use of ethylenediamine diacetoacetic in our patients did not depend on the fibrinolysis status. Therefore, our study could not evaluate the benefits of ethylenediamine diacetoacetic in patients with hyperfibrinolysis.

Thrombin can directly affect the conversion from fibrinogen to fibrin clots and acts on the coagulation cascade [25]. A previous study demonstrated that endoscopic injection of human thrombin was effective for gastric variceal bleeding [26]. Additionally, an Indian prospective study including 20 patients with gastric variceal bleeding showed that endoscopic injection of human thrombin was effective and the hemostatic successful rate was 100% [18]. However, in the two studies, endoscopic injection was the only mode of administration, and only gastric and ectopic varices were treated. By comparison, our patients received oral or local spray of lyophilizing thrombin powder, and a majority of our patients had esophageal varices.

Hemocoagulase, which is extracted from the venom of a snake, such as *Brothrops atrox* and *Agkistrodon blomhoffii ussurensis*, has a thrombin-like effect [27]. Recently, a randomized controlled trial demonstrated that the topical spray of hemocoagulase might not significantly increase the rate of hemostatic success as compared with traditional 8% norepinephrine (100% versus 94.0%, *P* = 0.060) [17]. By comparison, in our study, no hemostatic drug was employed as the control group, and intravenous infusion of hemocoagulase was the only mode of administration. Notably, compared with local spray, intravenous infusion can cause hypofibrinogenemia, which may aggravate bleeding. Indeed, this phenomenon has been observed in several case reports [28–30].

Recombinant factor VIIa (rFVIIa) is not sufficiently supported by the current evidence for the management of acute variceal bleeding [6]. Two randomized controlled trials assessed the efficacy of rFVIIa for acute variceal bleeding in patients with cirrhosis [19, 31]. The first study assessed 245 cirrhotic patients with AUGIB by assessing a composite endpoint, which consisted of failure to control bleeding within 24 hours, failure to prevent rebleeding between 24 hours and day 5, or death within 5 days. Compared with placebo, rFVIIa significantly improved the composite endpoint (8% versus 23%, *P* = 0.03) in the subgroup analysis of Child-Pugh B class and C patients with variceal bleeding, despite the overall analysis found that the endpoint was not significantly different between rFVIIa and placebo groups (14% versus 16%, *P* = 0.72). Then, the investigators further selected a total of 256 cirrhotic patients with Child-Pugh class B and C and variceal bleeding in a second study to evaluate the same endpoint. Compared with placebo, rFVIIa did not add any significant benefit (23% versus 20%, OR = 0.80, *P* = 0.37) and had a lower rate of the composite endpoint (13%). Our meta-analysis of the two trials suggested that the difference in the endpoint was not significant between placebo and rFVIIa groups. Therefore, the effect of rFVIIa in cirrhotic patients with AUGIB remains controversial [32].

Vitamin K participates into the formation of coagulation factors II, VII, IX, and X in the liver and is usually used as a supplementary intervention [33]. Cirrhosis reduces the ability of synthesizing vitamin K-dependent clotting factors [34]. But intravenous infusion of vitamin K is not recommended to correct the coagulation abnormalities in cirrhosis with bleeding [20]. Indeed, vitamin K failed to achieve a remarkable benefit in the reduction of INR in cirrhosis patients [35]. Similarly, our study also suggested that intravenous infusion of vitamin K brings no benefit for treating gastrointestinal bleeding in patients with cirrhosis.

Due to the retrospective nature of this study, several limitations should be acknowledged. First, not all patients had Child-Pugh and MELD scores due to missing laboratory data. But we conducted the subgroup analyses according to the Child-Pugh class and MELD score. Second, not all patients underwent endoscopy to determine the presence and severity of gastroesophageal varices. But we conducted the subgroup analysis according to the use of endoscopic variceal therapy. Third, different hemostatic drugs during hospitalization were often combined. Fourth, a decision on the use of hemostatic drugs was arbitrarily made by our physicians. But we attempted to conduct the PSM analysis by adjusting 15 confounding factors that are associated with patients' outcomes. Finally, some hemostatic drugs were domestic, such as ethylenediamine diacetoacetic. Other hemostatic drugs were traditional Chinese medicine, such as Yunnan Baiyao. Both of them were not available in the West. The new topical hemostatic powder represents a user-friendly and effective tool in the management of upper gastrointestinal bleeding during endoscopic therapy procedures [36]. However, it has not been available at our hospital.

In conclusion, the effect of hemostatic drugs on AUGIB in cirrhotic patients was unsatisfactory, because the use of hemostatic drugs did not decrease the 5-day rebleeding rate or the in-hospital mortality in cirrhotic patients with AUGIB. Notably, most of the rebleeding events occurred after the initial use of hemostatic drugs. Recent advances in the management of AUGIB should be acknowledged. Future studies should employ more recent data to validate our findings. Additionally, considering the limitations of our study, well-designed randomized controlled trials are still needed in future.

## Figures and Tables

**Table 1:** Overall analysis of the difference between hemostatic drugs and no hemostatic drugs group.

Variables	No. Pts	Overall	No. Pts	Hemostatic drug group	No. Pts	No hemostatic drug group	*P* value
Age (years)	982	56.01 (6.28-95.13) 56.45 ± 11.87	870	56.01 (6.28-95.13) 56.51 ± 11.76	112	55.45 (24.92-84.77) 56.04 ± 12.76	0.536
Sex (male) (%)	982	688 (70.10%)	870	608 (69.90%)	112	80 (71.40%)	0.737
Cancer (%)	982	189 (19.20%)	870	169 (19.40%)	112	20 (17.90%)	0.692
Liver cancer (%)	982	160 (16.30%)	870	142 (16.30%)	112	18 (16.10%)	0.946
Extrahepatic cancer (%)	982	29 (3.00%)	870	27 (3.10%)	112	2 (1.80%)	0.438
Clinical features of AUGIB (%)							
Hematemesis (%)	982	587 (59.80%)	870	541 (62.20%)	112	46 (41.10%)	<0.001
Melena (%)	982	770 (78.40%)	870	675 (77.60%)	112	95 (84.80%)	0.080
Hematemesis and melena (%)	982	375 (38.20%)	870	346 (39.80%)	112	29 (25.90%)	0.004
Etiology of liver diseases	982		870		112		0.305
HBV (%)	982	399 (40.60%)	870	359 (41.30%)	112	40 (35.70%)	0.260
HCV (%)	982	83 (8.50%)	870	75 (8.60%)	112	8 (7.10%)	0.597
Alcohol abuse (%)	982	354 (36.00%)	870	315 (36.20%)	112	39 (34.80%)	0.774
HBV+alcohol abuse (%)	982	106 (10.80%)	870	94 (10.80%)	112	12 (10.70%)	0.977
HCV+alcohol abuse (%)	982	18 (1.80%)	870	14 (1.60%)	112	4 (3.60%)	0.145
Other or unknown etiology (%)	982	186 (18.90%)	870	159 (18.30%)	112	27 (24.10%)	0.138
Endoscopic evaluation of EV (%)	982	563 (57.30%)	870	503 (57.80%)	112	60 (53.60%)	0.393
No EV (%)	982	34 (3.50%)	870	26 (3.00%)	112	8 (7.10%)	0.024
Mild EV (%)	982	27 (2.70%)	870	26 (3.00%)	112	1 (0.90%)	0.202
Moderate EV (%)	982	66 (6.70%)	870	60 (6.90%)	112	6 (5.40%)	0.540
Severe EV (%)	982	437 (44.50%)	870	392 (45.10%)	112	45 (40.20%)	0.328
Laboratory tests							
Red Blood Cell (10^12^/L)	973	2.54 (0.79-5.94) 2.62 ± 0.70	864	2.52 (0.79-5.49) 2.59 ± 0.69	109	2.81 (1.21-5.08) 2.81 ± 0.71	0.002
Hemoglobin (g/L)	974	72.00 (19.00-180.00) 75.10 ± 22.58	865	72.00 (19.00-180.00) 74.75 ± 22.47	109	75.00 (31.00-170.00) 77.88 ± 23.38	0.165
White blood cell (10^9^/L)	974	4.90 (0.80-46.10) 6.17 ± 4.75	865	4.90 (0.80-46.10) 6.19 ± 4.76	109	4.50 (1.10-30.70) 6.02 ± 4.69	0.658
Platelet (10^9^/L)	974	76.00 (9.00-842.00) 95.96 ± 77.81	865	75.00 (9.00-775.00) 93.75 ± 74.28	109	84.00 (17.00-842.00) 113.51 ± 100.29	0.010
Total bilirubin (*μ*mol/L)	964	20.70 (3.30-553.60) 31.34 ± 39.68	855	20.60 (3.30-553.60) 31.38 ± 40.46	109	22.60 (5.90-241.40) 31.04 ± 33.14	0.857
Albumin (g/L)	949	29.90 (9.60-49.30) 29.69 ± 6.75	841	29.60 (9.60-49.30) 29.43 ± 6.69	108	31.45 (13.60-48.40) 31.66 ± 6.94	0.002
Alanine aminotransferase (U/L)	962	24.00 (5.00-1064.00) 37.64 ± 61.52	853	24.00 (5.00-1064.00) 37.66 ± 62.94	109	24.00 (5.00-438.00) 37.40 ± 49.23	0.764
Aspartate aminotransferase (U/L)	962	32.00 (7.00-1487.00) 58.93 ± 117.01	853	32.00 (7.00-1487.00) 58.87 ± 117.48	109	31.00 (13.00-994.00) 59.39 ± 113.76	0.906
Alkaline phosphatase (U/L)	962	74.90 (1.30-889.00) 99.86 ± 88.33	853	73.00 (1.30-889.00) 97.68 ± 85.57	109	84.00 (28.00-868.00) 116.92 ± 106.48	0.002
Gamma-glutamyl transpeptidase (U/L)	962	40.00 (5.00-1168.00) 87.40 ± 126.42	853	39.00 (5.00-1168.00) 83.96 ± 119.53	109	48.00 (10.00-994.00) 114.36 ± 169.35	0.126
Blood urea nitrogen (mmol/L)	932	7.81 (1.54-55.01) 9.12 ± 5.99	827	7.94 (1.54-49.19) 9.28 ± 5.85	105	6.19 (2.22-55.01) 7.92 ± 6.90	<0.001
Serum creatinine (*μ*mol/L)	930	60.20 (20.00-1189.00) 72.69 ± 69.59	825	61.00 (20.00-1189.00) 72.43 ± 66.07	105	57.00 (28.00-919.00) 74.77 ± 93.11	0.154
Potassium (mmol/L)	952	4.07 (2.13-7.87) 4.11 ± 0.55	845	4.09 (2.13-7.87) 4.13 ± 0.57	107	4.00 (2.79-5.80) 4.00 ± 0.43	0.003
Sodium (mmol/L)	952	138.60 (83.00-160.80) 138.17 ± 5.14	845	138.70 (83.00-160.10) 138.22 ± 5.14	107	138.00 (118.70-160.80) 137.84 ± 5.21	0.146
Prothrombin time (seconds)	922	16.20 (10.80-62.80) 17.29 ± 4.82	816	16.30 (11.00-62.80) 17.44 ± 4.89	106	14.95 (10.80-40.90) 16.13 ± 4.17	<0.001
INR	920	1.31 (0.77-7.96) 1.45 ± 0.58	814	1.33 (0.79-7.96) 1.46 ± 0.59	106	1.18 (0.77-4.19) 1.32 ± 0.48	<0.001
Child-Pugh score	901	7.00 (5.00-15.00) 7.78 ± 2.00	798	7.00 (5.00-15.00) 7.81 ± 1.95	103	7.00 (5.00-14.00) 7.51 ± 2.30	0.187
Child-Pugh class A/B/C (%)	901	256 (28.40%)/476 (52.80%)/169 (18.80%)	798	215 (26.90%)/433 (54.30%)/150 (18.80%)	103	41 (39.80%)/43 (41.70%)/19 (18.40%)	0.018
MELD score	895	6.60 (-7.52-40.95) 7.59 ± 6.71	795	6.74 (-7.44-39.17) 7.76 ± 6.64	100	5.21 (-7.52-40.95) 6.24 ± 7.09	0.056
Vasoactive drugs (%)	982	892 (90.80%)	870	815 (93.70%)	112	77 (68.80%)	<0.001
Somatostatin (%)	982	814 (82.90%)	870	755 (86.80%)	112	59 (50.40%)	<0.001
Octreotide (%)	982	379 (38.60%)	870	332 (38.20%)	112	47 (42.00%)	0.436
Proton-pump inhibitors (%)	982	967 (98.50%)	870	864 (99.30%)	112	103 (92.00%)	<0.001
Red blood cell transfusion (%)	982	611 (62.20%)	870	561 (64.50%)	112	50 (44.60%)	<0.001
Antibiotics (%)	982	468 (47.70%)	870	416 (47.80%)	112	52 (46.40%)	0.782
5-day rebleeding (%)	981	163 (16.60%)	869	157 (18.10%)	112	6 (5.40%)	0.001
In-hospital death (%)	982	67 (6.80%)	870	62 (7.10%)	112	5 (4.50%)	0.293

Abbreviations: Pts: patients; HBV: hepatitis B virus; HCV: hepatitis C virus; AUGIB: acute upper gastrointestinal bleeding; INR: international normalized ratio; APTT: activated partial thromboplastin time; MELD: model for end-stage liver disease; EV: esophageal varices.

**Table 2:** PSM analysis of difference between hemostatic drugs and no hemostatic drug groups.

Variables	No. Pts	Hemostatic drugs group	No. Pts	No hemostatic drug group	*P* value
Age (years)	86	55.60 (30.37-89.23) 57.00 ± 11.39	86	55.78 (24.92-84.77) 55.65 ± 11.73	0.504
Sex (male) (%)	86	60 (69.80%)	86	62 (72.10%)	0.737
Cancer (%)	86	10 (11.60%)	86	16 (18.60%)	0.201
Liver cancer (%)	86	8 (9.30%)	86	15 (17.40%)	0.117
Extrahepatic cancer (%)	86	2 (2.30%)	86	1 (1.20%)	0.560
Clinical features of AUGIB (%)					
Hematemesis (%)	86	33 (38.40%)	86	36 (41.90%)	0.641
Melena (%)	86	72 (83.70%)	86	74 (86.00%)	0.670
Both hematemesis and melena (%)	86	19 (22.10%)	86	24 (27.90%)	0.379
Etiology of liver diseases					
HBV (%)	86	22 (25.60%)	86	32 (37.20%)	0.100
HCV (%)	86	5 (5.80%)	86	7 (8.10%)	0.549
Alcohol abuse (%)	86	33 (38.40%)	86	34 (39.50%)	0.876
HBV+alcohol abuse (%)	86	6 (7.00%)	86	11 (12.80%)	0.201
HCV+alcohol abuse (%)	86	3 (3.50%)	86	4 (4.70%)	0.700
Other or unknown etiology (%)	86	35 (40.70%)	86	29 (33.70%)	0.344
Endoscopic evaluation of EV (%)	86	53 (61.60%)	86	49 (57.00%)	0.535
No EV (%)	86	5 (5.80%)	86	7 (8.10%)	0.549
Mild EV (%)	86	3 (3.50%)	86	1 (1.20%)	0.312
Moderate EV (%)	86	6 (7.00%)	86	5 (5.80%)	0.755
Severe EV (%)	86	39 (45.30%)	86	36 (41.90%)	0.646
Laboratory tests					
Red Blood Cell (10^12^/L)	86	2.58 (0.93-5.07) 2.67 ± 0.73	86	2.68 (1.21-4.22) 2.71 ± 0.62	0.438
Hemoglobin (g/L)	86	73.00 (31.00-157.00) 78.64 ± 24.96	86	73.50 (42.00-122.00) 74.99 ± 19.85	0.608
White blood cell (10^9^/L)	86	4.05 (1.00-25.20) 5.35 ± 4.29	86	4.20 (1.10-30.70) 5.70 ± 4.62	0.400
Platelet (10^9^/L)	86	70.50 (9.00-775.00) 97.90 ± 96.51	86	82.00 (17.00-842.00) 111.50 ± 105.11	0.103
Total bilirubin (*μ*mol/L)	86	20.10 (4.80-553.60) 35.42 ± 64.87	86	23.25 (5.90-241.40) 30.03 ± 30.64	0.968
Albumin (g/L)	85	30.90 (17.20-49.30) 30.60 ± 6.47	85	31.20 (13.60-48.00) 31.58 ± 7.12	0.337
Alanine aminotransferase (U/L)	86	26.00 (6.00-184.00) 32.23 ± 24.92	86	23.00 (5.00-438.00) 37.58 ± 53.46	0.548
Aspartate aminotransferase (U/L)	86	35.50 (8.00-228.00) 49.35 ± 42.70	86	30.50 (13.00-994.00) 60.43 ± 125.71	0.218
Alkaline phosphatase (U/L)	86	76.30 (36.00-707.00) 114.00 ± 102.19	86	92.50 (28.00-450.00) 104.13 ± 61.66	0.381
Gamma-glutamyl transpeptidase (U/L)	86	55.50 (8.00-1168.00) 131.82 ± 209.08	86	49.50 (10.00-994.00) 103.97 ± 154.16	0.454
Blood urea nitrogen (mmol/L)	86	7.37 (2.07-24.92) 8.54 ± 4.91	86	6.20 (2.22-55.01) 8.04 ± 7.18	0.177
Serum Creatinine (*μ*mol/L)	86	55.00 (24.00-449.00) 65.36 ± 49.07	86	57.00 (28.00-919.00) 72.37 ± 97.01	0.565
Potassium (mmol/L)	85	4.07 (2.13-5.48) 4.08 ± 0.55	85	4.00 (2.79-5.80) 4.02 ± 0.45	0.430
Sodium (mmol/L)	85	139.40 (128.90-147.30) 139.08 ± 3.77	85	137.60 (122.60-146.50) 137.51 ± 4.47	0.023
Prothrombin time (seconds)	86	15.60 (12.90-47.00) 16.71 ± 4.44	86	15.25 (10.80-40.90) 16.37 ± 4.44	0.280
INR	86	1.25 (0.97-5.21) 1.38 ± 0.53	86	1.20 (0.77-4.19) 1.34 ± 0.51	0.219
Child-Pugh score	86	7.00 (5.00-14.00) 7.63 ± 2.05	86	7.00 (5.00-14.00) 7.52 ± 2.33	0.460
Child-Pugh class A/B/C (%)	86	29 (33.70%)/42 (48.80%)/15 (17.50%)	86	35 (40.70%)/35 (40.70%)/16 (18.60%)	0.585
MELD score	86	5.27 (-6.44-32.06) 6.28 ± 6.84	86	5.09 (-7.52-40.95) 6.17 ± 7.05	0.861
Endoscopic variceal treatment (%)	86	45 (52.30%)	86	38 (44.20%)	0.285
Vasoactive drugs (%)	86	67 (77.90%)	86	65 (75.60%)	0.718
Somatostatin (%)	86	60 (69.80%)	86	49 (57.70%)	0.082
Octreotide (%)	86	31 (36.00%)	86	40 (46.50%)	0.163
Proton-pump inhibitors (%)	86	83 (96.50%)	86	84 (97.70%)	0.650
Antibiotics (%)	86	41 (47.70%)	86	43 (50.00%)	0.760
Red blood cell transfusion (%)	86	47 (54.70%)	86	44 (51.20%)	0.647
5-day rebleeding (%)	86	13 (15.10%)	86	5 (5.80%)	0.046
In-hospital death (%)	86	6 (7.00%)	86	3 (3.50%)	0.304

Abbreviations: Pts: patients; HBV: hepatitis B virus; HCV: hepatitis C virus; AUGIB: acute upper gastrointestinal bleeding; INR: international normalized ratio; APTT: activated partial thromboplastin time; MELD: model for end-stage liver disease; EV: esophageal varices.

## Data Availability

The datasets generated during and/or analyzed during the current study are available from the corresponding author on reasonable request.

## Abbreviations

AUGIB: Acute upper gastrointestinal bleeding
INR: International normalized ratio
MELD: Model for end-stage liver disease
HCC: Hepatocellular carcinoma
PPIs: Proton pump inhibitors
PSM: Propensity score matching
rFVIIa: Recombinant factor VIIa
OR: Odds ratio

## Supplementary Materials

Supplementary Materials Supplementary Table 1: Mechanisms and indications of different hemostatic drugs. Supplementary Table 2: PSM analysis of difference between ethylenediamine diacetoacetic and no hemostatic drug groups. Supplementary Table 3: PSM analysis of difference between lyophilizing thrombin powder and no hemostatic drug groups. Supplementary Table 4: PSM analysis of difference between white-browed snake venom hemocoagulase and no hemostatic drug groups. Supplementary Table 5: PSM analysis of difference between snake venom hemocoagulase and no hemostatic drug groups. Supplementary Table 6: PSM analysis of difference between Yunnan Baiyao and no hemostatic drug groups. Supplementary Table 7: PSM analysis of difference between norepinephrine and no hemostatic drug groups. Supplementary Table 8: PSM analysis of difference between carbazochrome sodium sulfonate and no hemostatic drug groups. Supplementary Table 9: PSM analysis of difference between vitamin K and no hemostatic drug groups.
