# Supplementary material for: No Benefit of Hemostatic Drugs on Acute Upper Gastrointestinal Bleeding in Cirrhosis
Source: Biomed Res Int. 2020 Jun 26;2020:4097170. doi: 10.1155/2020/4097170 (PMC7336197; doi:10.1155/2020/4097170)
Supplement: Supplementary Materials — Supplementary Table 1: Mechanisms and indications of different hemostatic drugs. Supplementary Table 2: PSM analysis of difference between ethylenediamine diacetoacetic and no hemostatic drug groups. Supplementary Table 3: PSM analysis of difference between lyophilizing thrombin powder and no hemostatic drug groups. Supplementary Table 4: PSM analysis of difference between white-browed snake venom hemocoagulase and no hemostatic drug groups. Supplementary Table 5: PSM analysis of difference between snake venom hemocoagulase and no hemostatic drug groups. Supplementary Table 6: PSM analysis of difference between Yunnan Baiyao and no hemostatic drug groups. Supplementary Table 7: PSM analysis of difference between norepinephrine and no hemostatic drug groups. Supplementary Table 8: PSM analysis of difference between carbazochrome sodium sulfonate and no hemostatic drug groups. Supplementary Table 9: PSM analysis of difference between vitamin K and no hemostatic drug groups. [file 4097170.f1.doc]

**Supplementary Table 1. Mechanisms and indications of different hemostatic drugs**

| **Drug** | **Mechanisms** | **Indications** |
| --- | --- | --- |
| Vitamin K | Control the formation of coagulation factors II, VII, IX and X, regulates protein C and protein S through the liver. | Lacking of vitamin K-induced bleeding. |
| Ethylenediamine diacetoacetic | Inhibit the formation of plasminogen complex; promote platelet release of active substances; reduce capillary permeability. | Bleeding caused by hyperfibrinolysis. |
| Lyophilizing thrombin powder | Promote the conversion of fibrinogen to fibrin; enhance local platelet aggregation and release; promote epithelial cell production. | Bleeding from capillaries, small blood vessels and organs. Oral or topical spray. |
| Yunnan Baiyao* | Promote platelet aggregation and enhance platelet activation. | Resolving phlegm and relieving blood, for vomiting blood, hemoptysis, blood in the stool, ulcer bleeding. |
| Norepinephrine | Antagonist of alpha receptor, contractile arterioles and venules. | Bleeding of local gastric mucosa hemostatic in upper gastrointestinal. |
| Carbazochrome sodium sulfonate | Reduce capillary fragility and increase capillary resistance to damage. | Bleeding of upper digestive, urinary system, respiratory system. |
| Hemocoagulase | Degrade fibrinogen to form fibrin monomer; promote thrombin generation, activate factor Xa; act on XIIIa to stabilize fibrin. | Various medical situations where it is necessary to reduce bleeding. |

*Traditional Chinese medicine containing notoginseng (Panax pseudoginseng) root, borneol (Dryobalanops aromatica) crystal, Boea Clarkean (Boea clarkeana) entire plant, Inula Copp (Inula coppa) root, Complanatum (Lycopodium complanatum) rhizome, Chinese Yam (Dioscorea opposita) rhizome, galanga (Alpina offcinarum), cranebill (Erodium stephanianum) aerial parts, camphor (Cinnamonmum camphora) crystal extracts, and peppermint (Menta haplocalyx) leaves as active ingredients.

**Supplementary Table 2. PSM analysis of difference between ethylenediamine diacetoacetic and no hemostatic drug groups**

| **Variables** | **No. Pts** | **Ethylenediamine diacetoacetic group** | **No. Pts** | **No hemostatic drug group** | **P value** |
| --- | --- | --- | --- | --- | --- |
| **Age (years)** | 75 | 54.72 (30.08-89.23)  55.86±12.40 | 75 | 54.34 (24.92-84.77)  55.85±12.13 | 0.966 |
| **Sex (male) (%)** | 75 | 51 (68.00%) | 75 | 54 (72.00%) | 0.593 |
| **Cancer (%)** | 75 | 20 (26.70%) | 75 | 13 (17.30%) | 0.168 |
| Liver cancer (%) | 75 | 19 (25.30%) | 75 | 12 (16.00%) | 0.158 |
| Extrahepatic cancer (%) | 75 | 1 (1.30%) | 75 | 1 (1.30%) | 1.000 |
| **Clinical features of AUGIB (%)** |  |  |  |  |  |
| Hematemesis (%) | 75 | 36 (48.00%) | 75 | 33 (44.00%) | 0.623 |
| Melena (%) | 75 | 64 (85.30%) | 75 | 63 (84.00%) | 0.821 |
| Both hematemesis and melena (%) | 75 | 25 (33.30%) | 75 | 21 (28.00%) | 0.479 |
| **Etiology of Liver Diseases** | 75 |  | 75 |  | 0.924 |
| HBV (%) | 75 | 32 (42.70%) | 75 | 28 (37.30%) | 0.505 |
| HCV (%) | 75 | 7 (9.30%) | 75 | 5 (6.70%) | 0.547 |
| Alcohol Abuse (%) | 75 | 22 (29.30%) | 75 | 25 (33.30%) | 0.597 |
| HBV+Alcohol Abuse (%) | 75 | 6 (8.00%) | 75 | 7 (9.30%) | 0.772 |
| HCV+Alcohol Abuse (%) | 75 | 2 (2.70%) | 75 | 2 (2.70%) | 1.000 |
| Drug Related Liver Diseases (%) | 75 | 5 (6.70%) | 75 | 5 (6.70%) | 1.000 |
| Autoimmune Liver Diseases (%) | 75 | 4 (5.30%) | 75 | 6 (8.00%) | 0.513 |
| Other or Unknown Etiology (%) | 75 | 14 (18.70%) | 75 | 16 (21.30%) | 0.683 |
| **Endoscopic evaluation of EV (%)** | 75 | 33 (44.00%) | 75 | 41 (54.70%) | 0.191 |
| No EV (%) | 75 | 2 (2.70%) | 75 | 6 (8.00%) | 0.146 |
| Mild EV (%) | 75 | 3 (4.00%) | 75 | 1 (1.30%) | 0.311 |
| Moderate EV (%) | 75 | 4 (5.30%) | 75 | 4 (5.30%) | 1.000 |
| Severe EV (%) | 75 | 24 (32.00%) | 75 | 30 (40.00%) | 0.307 |
| **Laboratory Tests** |  |  |  |  |  |
| Red Blood Cell (10^12^/L) | 75 | 2.60 (0.93-4.38)  2.67±0.79 | 75 | 2.68 (1.21-4.22)  2.71±0.63 | 0.434 |
| Hemoglobin (g/L) | 75 | 74.00 (29.00-145.00)  78.37±25.70 | 75 | 72.00 (43.00-122.00)  74.43±19.52 | 0.474 |
| White Blood Cell (10^9^/L) | 75 | 4.60 (0.90-27.30)  6.26±5.39 | 75 | 4.50 (1.10-30.70)  6.01±4.85 | 0.897 |
| Platelet (10^9^/L) | 75 | 87.00 (14.00-587.00)  102.19±79.46 | 75 | 87.00 (17.00-842.00)  117.76±111.98 | 0.488 |
| Total Bilirubin (μmol/L) | 75 | 19.80 (4.90-553.60)  36.01±76.73 | 75 | 21.00 (5.90-241.40)  29.66±32.49 | 0.814 |
| Albumin (g/L) | 74 | 30.20 (11.80-47.20)  30.41±6.82 | 74 | 31.30 (13.60-48.00)  31.50±7.17 | 0.419 |
| Alanine Aminotransferase (U/L) | 75 | 25.00 (6.00-197.00)  32.40±26.94 | 75 | 22.00 (5.00-438.00)  37.51±56.22 | 0.403 |
| Aspartate Aminotransferase (U/L) | 75 | 32.00 (10.00-319.00)  44.16±42.63 | 75 | 31.00 (13.00-994.00)  58.35±131.65 | 0.722 |
| Alkaline Phosphatase (U/L) | 75 | 74.00 (29.00-388.00)  100.94±75.48 | 75 | 80.00 (28.00-391.00)  100.95±61.39 | 0.288 |
| Gamma-Glutamyl Transpeptidase (U/L) | 75 | 40.00 (5.00-702.00)  84.51±126.42 | 75 | 42.00 (10.00-994.00)  100.84±165.55 | 0.842 |
| Blood Urea Nitrogen (mmol/L) | 75 | 7.12 (2.03-25.86)  8.00±4.30 | 75 | 6.20 (2.22-55.01)  8.31±7.58 | 0.419 |
| Serum Creatinine (μmol/L) | 75 | 53.00 (29.00-249.00)  61.13±31.60 | 75 | 58.00 (28.00-919.00)  75.24±103.51 | 0.144 |
| Potassium (mmol/L) | 74 | 4.14 (3.13-6.85)  4.13±0.52 | 75 | 4.01(2.98-5.80)  4.04±0.44 | 0.201 |
| Sodium (mmol/L) | 74 | 137.85 (121.30-146.20)  137.63±4.87 | 75 | 138.50 (122.60-146.50)  137.92±4.41 | 0.786 |
| Prothrombin Time (seconds) | 75 | 16.20 (12.70-30.40)  16.92±3.33 | 75 | 15.20 (10.80-40.90)  16.56±4.66 | 0.118 |
| INR | 75 | 1.31 (0.94-2.87)  1.40±0.36 | 75 | 1.20 (0.77-4.19)  1.37±0.54 | 0.092 |
| APTT (seconds) | 75 | 40.70 (19.50-64.80)  41.25±7.54 | 75 | 41.50 (28.40-66.90)  42.02±7.54 | 0.601 |
| **Child-Pugh Score** | 75 | 7.00 (5.00-14.00)  7.41±1.88 | 75 | 7.00 (5.00-14.00)  7.44±2.36 | 0.506 |
| **Child-Pugh Class A/B/C (%)** | 75 | 24 (32.00%)/42  (56.00%)/9 (12.00%) | 75 | 33 (44.00%)/28  (37.30%)/14 (18.70%) | 0.070 |
| **MELD Score** | 75 | 5.14 (-6.50-39.17)  6.11±6.56 | 75 | 5.06 (-7.52-40.95)  6.39±7.33 | 0.882 |
| **Endoscopic** **variceal treatment (%)** | 75 | 33 (44.00%) | 75 | 32 (42.70%) | 0.869 |
| **Vasoactive drugs (%)** | 75 | 63 (84.00%) | 75 | 61 (81.30%) | 0.666 |
| Somatostatin (%) | 75 | 57 (76.00%) | 75 | 45 (60.00%) | ***0.036*** |
| Octreotide (%) | 75 | 30 (40.00%) | 75 | 37 (49.30%) | 0.250 |
| **Proton-pump inhibitor (%)** | 75 | 73 (97.30%) | 75 | 74 (98.70%) | 0.560 |
| **Antibiotics (%)** | 75 | 40 (53.30%) | 75 | 38 (50.70%) | 0.744 |
| **Red blood cell transfusion (%)** | 75 | 35 (46.70%) | 75 | 49 (52.00%) | 0.514 |
| **5-day rebleeding (%)** | 75 | 10 (13.30%) | 75 | 4 (5.30%) | 0.092 |
| **In-hospital death (%)** | 75 | 4 (5.30%) | 75 | 3 (4.00%) | 0.699 |

**Abbreviations:** Pts: Patients; HBV: Hepatic B Virus; HCV: Hepatic C Virus; AUGIB: Acute Upper Gastrointestinal Bleeding; INR: International Standardization Ratio; APTT: Activated Partial Thromboplastin Time; MELD: Model for End-stage Liver Disease; EV: Esophageal Varices.

**Supplementary Table 3. PSM analysis of difference between lyophilizing thrombin powder and no hemostatic drug groups**

| **Variables** | **No. Pts** | **Lyophilizing thrombin powder group** | **No. Pts** | **No hemostatic drug group** | **P value** |
| --- | --- | --- | --- | --- | --- |
| **Age (years)** | 70 | 55.23 (30.08-83.38)  55.43±11.00 | 70 | 54.64 (37.80-84.77)  55.88±10.17 | 0.819 |
| **Sex (male) (%)** | 70 | 44 (62.90%) | 70 | 51 (72.90%) | 0.205 |
| **Cancer (%)** | 70 | 11 (15.70%) | 70 | 13 (18.60%) | 0.654 |
| Liver cancer (%) | 70 | 9 (12.90%) | 70 | 12 (17.10%) | 0.478 |
| Extrahepatic cancer (%) | 70 | 2 (2.90%) | 70 | 1 (1.40%) | 0.559 |
| **Clinical features of AUGIB (%)** |  |  |  |  |  |
| Hematemesis (%) | 70 | 36 (51.40%) | 70 | 31 (44.30%) | 0.398 |
| Melena (%) | 70 | 59 (84.30%) | 70 | 58 (82.90%) | 0.820 |
| Both hematemesis and melena (%) | 70 | 25 (35.70%) | 70 | 19 (27.10%) | 0.275 |
| **Etiology of Liver Diseases** | 70 |  | 70 |  | 0.688 |
| HBV (%) | 70 | 33 (47.10%) | 70 | 28 (40.00%) | 0.394 |
| HCV (%) | 70 | 8 (11.40%) | 70 | 5 (7.10%) | 0.382 |
| Alcohol Abuse (%) | 70 | 23 (32.90%) | 70 | 29 (41.40%) | 0.294 |
| HBV+Alcohol Abuse (%) | 70 | 7 (10.00%) | 70 | 10 (14.30%) | 0.438 |
| HCV+Alcohol Abuse (%) | 70 | 2 (2.90%) | 70 | 2 (2.90%) | 1.000 |
| Drug Related Liver Diseases (%) | 70 | 2 (2.90%) | 70 | 2 (2.90%) | 1.000 |
| Autoimmune Liver Diseases (%) | 70 | 2 (2.90%) | 70 | 7 (10.00%) | 0.085 |
| Other or Unknown Etiology (%) | 70 | 13 (18.60%) | 70 | 12 (17.10%) | 0.825 |
| **Endoscopic evaluation of EV (%)** | 70 | 33 (47.10%) | 70 | 43 (61.40%) | 0.090 |
| No EV (%) | 70 | 4 (5.70%) | 70 | 5 (7.10%) | 0.730 |
| Mild EV (%) | 70 | 0 (0.00%) | 70 | 1 (1.40%) | 0.316 |
| Moderate EV (%) | 70 | 5 (7.10%) | 70 | 5 (7.10%) | 1.000 |
| Severe EV (%) | 70 | 24 (34.30%) | 70 | 32 (45.70%) | 0.168 |
| **Laboratory Tests** |  |  |  |  |  |
| Red Blood Cell (10^12^/L) | 69 | 2.74 (1.27-4.33)  2.80±0.72 | 70 | 2.82 (1.21-4.22)  2.80±0.62 | 0.975 |
| Hemoglobin (g/L) | 69 | 76.00 (36.00-136.00)  78.10±24.25 | 70 | 76.00 (42.00-119.00)  77.49±19.23 | 0.985 |
| White Blood Cell (10^9^/L) | 69 | 4.60 (0.90-27.30)  6.26±5.39 | 70 | 4.40 (1.10-24.70)  5.36±3.71 | 0.970 |
| Platelet (10^9^/L) | 69 | 87.00 (14.00-587.00)  102.19±79.46 | 70 | 83.00 (17.00-293.00)  97.93±51.70 | 0.117 |
| Total Bilirubin (μmol/L) | 70 | 21.80 (4.10-117.50)  26.47±19.08 | 70 | 24.90 (5.90-105.30)  29.84±21.26 | 0.442 |
| Albumin (g/L) | 70 | 32.50 (12.40-47.20)  31.33±6.81 | 69 | 31.10 (13.60-48.00)  31.54±7.31 | 0.796 |
| Alanine Aminotransferase (U/L) | 70 | 20.50 (7.00-169.00)  28.99±25.34 | 70 | 24.00 (7.00-208.00)  35.10±32.07 | 0.098 |
| Aspartate Aminotransferase (U/L) | 70 | 29.50 (11.00-343.00)  39.94±44.45 | 70 | 31.50 (14.00-634.00)  51.87±76.44 | ***0.037*** |
| Alkaline Phosphatase (U/L) | 70 | 68.50 (33.50-688.00)  102.74±100.08 | 70 | 88.00 (38.70-450.00)  111.98±79.68 | ***0.038*** |
| Gamma-Glutamyl Transpeptidase (U/L) | 70 | 35.00 (6.00-589.00)  69.03±90.32 | 70 | 52.00 (10.00-708.00)  117.30±160.20 | 0.060 |
| Blood Urea Nitrogen (mmol/L) | 70 | 7.11 (2.24-19.83)  8.03±4.03 | 70 | 6.14 (2.22-41.82)  7.27±5.15 | 0.128 |
| Serum Creatinine (μmol/L) | 70 | 56.50 (20.00-132.00)  57.29±17.62 | 70 | 55.150 (28.00-919.00)  72.53±105.72 | 0.709 |
| Potassium (mmol/L) | 69 | 4.08 (2.90-5.58)  4.14±0.56 | 70 | 4.01 (2.79-5.09)  4.01±0.43 | 0.309 |
| Sodium (mmol/L) | 69 | 138.60 (118.30-146.20)  137.57±5.37 | 70 | 137.75 (122.60-146.50)  137.62±4.47 | 0.727 |
| Prothrombin Time (seconds) | 70 | 15.75 (12.00-30.40)  16.31±2.96 | 70 | 15.35 (11.30-36.60)  16.33±3.78 | 0.524 |
| INR | 70 | 1.25 (0.92-2.99)  1.33±0.33 | 70 | 1.22 (0.82-3.73)  1.34±0.43 | 0.449 |
| APTT (seconds) | 70 | 40.35 (27.30-56.70)  40.24±6.41 | 70 | 40.75 (30.20-66.90)  41.81±6.80 | 0.213 |
| **Child-Pugh Score** | 70 | 7.00 (5.00-12.00)  7.27±1.51 | 70 | 7.00 (5.00-13.00)  7.57±2.35 | 0.992 |
| **Child-Pugh Class A/B+C (%)** | 70 | 22 (31.40%)/48 (68.60%) | 70 | 29 (41.40%)/41 (58.60%) | 0.219 |
| **MELD Score** | 70 | 5.25 (-3.37-20.01)  5.35±4.44 | 70 | 5.25 (-7.52-27.43)  6.29±6.07 | 0.412 |
| **Endoscopic** **variceal treatment (%)** | 70 | 33 (44.00%) | 70 | 32 (42.70%) | 0.869 |
| **Vasoactive drugs (%)** | 70 | 62 (88.60%) | 70 | 62 (88.60%) | 1.000 |
| Somatostatin (%) | 70 | 56 (80.00%) | 70 | 46 (65.70%) | 0.057 |
| Octreotide (%) | 70 | 24 (34.30%) | 70 | 41 (58.60%) | ***0.004*** |
| **Proton-pump inhibitor (%)** | 70 | 70 (100.00%) | 70 | 70 (100.00%) | NA |
| **Antibiotics (%)** | 70 | 38 (54.30%) | 70 | 36 (51.40%) | 0.735 |
| **Red blood cell transfusion (%)** | 70 | 35 (50.00%) | 70 | 35 (50.00%) | 1.000 |
| **5-day rebleeding (%)** | 70 | 10 (14.30%) | 70 | 4 (5.70%) | 0.091 |
| **In-hospital death (%)** | 70 | 2 (2.90%) | 70 | 2 (2.90%) | 1.000 |

**Abbreviations:** Pts: Patients; HBV: Hepatic B Virus; HCV: Hepatic C Virus; AUGIB: Acute Upper Gastrointestinal Bleeding; INR: International Standardization Ratio; APTT: Activated Partial Thromboplastin Time; MELD: Model for End-stage Liver Disease; EV: Esophageal Varices; NA: Not Available.

**Supplementary Table 4. PSM analysis of difference between white-browed snake venom hemocoagulase and no hemostatic drug groups**

| **Variables** | **No. Pts** | **White-browed snake venom hemocoagulase group** | **No. Pts** | **No hemostatic drug group** | **P value** |
| --- | --- | --- | --- | --- | --- |
| **Age (years)** | 64 | 54.28 (30.08-82.72)  54.27±10.83 | 64 | 54.24 (24.92-83.67)  55.40±12.90 | 0.634 |
| **Sex (male) (%)** | 64 | 47 (73.40%) | 64 | 46 (71.90%) | 0.843 |
| **Cancer (%)** | 64 | 12 (18.80%) | 64 | 11 (17.20%) | 0.818 |
| Liver cancer (%) | 64 | 12 (18.80%) | 64 | 10 (15.60%) | 0.639 |
| Extrahepatic cancer (%) | 64 | 0 (0.00%) | 64 | 1 (1.60%) | 0.315 |
| **Clinical features of AUGIB (%)** |  |  |  |  |  |
| Hematemesis (%) | 64 | 27 (42.20%) | 64 | 31 (48.40%) | 0.478 |
| Melena (%) | 64 | 56 (87.50%) | 64 | 54 (84.40%) | 0.611 |
| Both hematemesis and melena (%) | 64 | 19 (29.70%) | 64 | 21 (32.80%) | 0.703 |
| **Etiology of Liver Diseases** | 64 |  | 64 |  | 0.275 |
| HBV (%) | 64 | 29 (45.30%) | 64 | 18 (28.10%) | ***0.044*** |
| HCV (%) | 64 | 3 (4.70%) | 64 | 5 (7.80%) | 0.465 |
| Alcohol Abuse (%) | 64 | 22 (34.40%) | 64 | 23 (35.90%) | 0.853 |
| HBV+Alcohol Abuse (%) | 64 | 9 (14.10%) | 64 | 5 (7.80%) | 0.257 |
| HCV+Alcohol Abuse (%) | 64 | 1 (1.60%) | 64 | 2 (3.10%) | 0.559 |
| Drug Related Liver Diseases (%) | 64 | 2 (3.10%) | 64 | 4 (6.20%) | 0.403 |
| Autoimmune Liver Diseases (%) | 64 | 4 (6.20%) | 64 | 4 (6.20%) | 1.000 |
| Other or Unknown Etiology (%) | 64 | 14 (21.90%) | 64 | 18 (28.10%) | 0.414 |
| **Endoscopic evaluation of EV (%)** | 64 | 33 (47.10%) | 64 | 43 (61.40%) | 0.090 |
| No EV (%) | 64 | 2 (3.10%) | 64 | 6 (9.40%) | 0.144 |
| Mild EV (%) | 64 | 1 (1.60%) | 64 | 1 (1.60%) | 1.000 |
| Moderate EV (%) | 64 | 5 (7.80%) | 64 | 5 (7.80%) | 1.000 |
| Severe EV (%) | 64 | 25 (39.10%) | 64 | 29 (45.30%) | 0.474 |
| **Laboratory Tests** |  |  |  |  |  |
| Red Blood Cell (10^12^/L) | 64 | 2.73 (1.25-4.33)  2.71±0.74 | 64 | 2.62 (1.21-3.96)  2.69±0.63 | 0.883 |
| Hemoglobin (g/L) | 64 | 81.50 (29.00-137.00)  78.98±25.41 | 64 | 72.50 (31.00-119.00)  74.11±19.91 | 0.255 |
| White Blood Cell (10^9^/L) | 64 | 4.75 (1.00-29.10)  5.76±4.54 | 64 | 4.40 (1.10-30.70)  5.93±4.64 | 0.714 |
| Platelet (10^9^/L) | 64 | 80.00 (24.00-235.00)  88.67±42.78 | 64 | 88.00 (32.00-842.00)  120.77±119.34 | 0.187 |
| Total Bilirubin (μmol/L) | 64 | 20.30 (4.10-102.70)  25.59±19.60 | 64 | 23.25 (5.90-241.40)  33.15±39.79 | 0.668 |
| Albumin (g/L) | 62 | 30.35 (12.40-47.20)  30.42±6.49 | 64 | 31.15 (20.20-48.00)  31.60±6.33 | 0.416 |
| Alanine Aminotransferase (U/L) | 64 | 24.50 (8.00-234.00)  35.47±32.44 | 64 | 25.50 (5.00-438.00)  39.48±56.98 | 0.749 |
| Aspartate Aminotransferase (U/L) | 64 | 31.50 (15.00-337.00)  48.33±48.80 | 64 | 31.00 (13.00-994.00)  59.33±125.20 | 0.909 |
| Alkaline Phosphatase (U/L) | 64 | 77.00 (34.00-388.00)  95.43±67.68 | 64 | 79.50 (28.00-450.00)  114.62±91.49 | 0.178 |
| Gamma-Glutamyl Transpeptidase (U/L) | 64 | 48.50 (9.00-537.00)  85.21±93.29 | 64 | 47.50 (10.00-994.00)  117.59±183.72 | 0.987 |
| Blood Urea Nitrogen (mmol/L) | 64 | 7.64 (2.47-28.11)  8.56±4.78 | 64 | 6.31 (2.22-55.01)  8.55±8.17 | 0.207 |
| Serum Creatinine (μmol/L) | 64 | 61.50 (32.00-250.00)  63.83±29.47 | 64 | 60.50 (28.00-919.00)  77.30±110.37 | 0.873 |
| Potassium (mmol/L) | 64 | 4.10 (2.13-5.17)  4.04±0.46 | 63 | 4.00 (2.79-5.80)  4.03±0.48 | 0.335 |
| Sodium (mmol/L) | 64 | 139.15 (129.90-147.30)  138.74±3.69 | 63 | 137.60 (122.60-146.50)  137.32±4.54 | 0.727 |
| Prothrombin Time (seconds) | 64 | 15.85 (12.30-23.10)  16.47±2.68 | 64 | 14.86 (11.30-40.90)  15.99±4.08 | 0.065 |
| INR | 64 | 1.24 (0.94-2.10)  1.33±0.29 | 64 | 1.18 (0.82-4.19)  1.30±0.46 | 0.158 |
| APTT (seconds) | 64 | 39.30 (27.40-67.10)  40.03±6.37 | 64 | 41.50 (30.20-57.70)  41.73±6.30 | 0.089 |
| **Child-Pugh Score** | 64 | 7.00 (5.00-12.00)  7.31±1.64 | 64 | 7.00 (5.00-14.00)  7.45±2.25 | 0.786 |
| **Child-Pugh Class A/B/C (%)** | 64 | 21 (32.80%)/37  (57.80%)/6 (9.40%) | 64 | 26 (40.60%)/28  (43.80%)/10 (15.60%) | 0.249 |
| **MELD Score** | 64 | 5.36 (-6.44-19.85)  6.02±5.23 | 64 | 5.71 (-3.92-40.95)  6.41±6.72 | 0.943 |
| **Endoscopic** **variceal treatment (%)** | 64 | 31 (48.40%) | 64 | 31 (48.40%) | 1.000 |
| **Vasoactive drugs (%)** | 64 | 51 (79.70%) | 64 | 48 (75.00%) | 0.526 |
| Somatostatin (%) | 64 | 47 (73.40%) | 64 | 37 (57.80%) | 0.063 |
| Octreotide (%) | 64 | 18 (28.10%) | 64 | 29 (45.30%) | ***0.044*** |
| **Proton-pump inhibitor (%)** | 64 | 62 (96.90%) | 64 | 62 (96.90%) | 1.000 |
| **Antibiotics (%)** | 64 | 23 (35.90%) | 64 | 34 (53.10%) | 0.050 |
| **Red blood cell transfusion (%)** | 64 | 28 (43.80%) | 64 | 36 (56.20%) | 0.157 |
| **5-day rebleeding (%)** | 64 | 8 (12.50%) | 64 | 3 (4.70%) | 0.115 |
| **In-hospital death (%)** | 64 | 2 (3.10%) | 64 | 2 (3.10%) | 1.000 |

**Abbreviations:** Pts: Patients; HBV: Hepatic B Virus; HCV: Hepatic C Virus; AUGIB: Acute Upper Gastrointestinal Bleeding; INR: International Standardization Ratio; APTT: Activated Partial Thromboplastin Time; MELD: Model for End-stage Liver Disease; EV: Esophageal Varices.

**Supplementary Table 5. PSM analysis of difference between snake venom hemocoagulase and no hemostatic drug groups**

| **Variables** | **No. Pts** | **Snake venom hemocoagulase group** | **No. Pts** | **No hemostatic drug group** | **P value** |
| --- | --- | --- | --- | --- | --- |
| **Age (years)** | 31 | 56.02 (31.29-71.08)  54.97±9.61 | 31 | 52.75 (29.85-82.51)  54.74±12.31 | 0.559 |
| **Sex (male) (%)** | 31 | 21 (67.70%) | 31 | 22 (71.00%) | 0.783 |
| **Cancer (%)** | 31 | 11 (35.50%) | 31 | 4 (12.90%) | ***0.038*** |
| Liver cancer (%) | 31 | 9 (29.00%) | 31 | 4 (12.90%) | 0.119 |
| Extrahepatic cancer (%) | 31 | 2 (6.50%) | 31 | 0 (0.00%) | 0.151 |
| **Clinical features of AUGIB (%)** |  |  |  |  |  |
| Hematemesis (%) | 31 | 17 (54.80%) | 31 | 16 (51.60%) | 0.799 |
| Melena (%) | 31 | 22 (71.00%) | 31 | 28 (90.30%) | 0.054 |
| Both hematemesis and melena (%) | 31 | 8 (25.80%) | 31 | 13 (41.90%) | 0.180 |
| **Etiology of Liver Diseases** | 31 |  | 31 |  | 0.824 |
| HBV (%) | 31 | 16 (51.60%) | 31 | 13 (41.90%) | 0.445 |
| HCV (%) | 31 | 3 (9.70%) | 31 | 2 (6.50%) | 0.641 |
| Alcohol Abuse (%) | 31 | 10 (32.30%) | 31 | 9 (29.00%) | 0.783 |
| HBV+Alcohol Abuse (%) | 31 | 6 (19.40%) | 31 | 4 (12.90%) | 0.490 |
| HCV+Alcohol Abuse (%) | 31 | 1 (3.20%) | 31 | 0 (0.00%) | 0.313 |
| Drug Related Liver Diseases (%) | 31 | 1 (3.20%) | 31 | 1 (3.20%) | 1.000 |
| Autoimmune Liver Diseases (%) | 31 | 1 (3.20%) | 31 | 3 (9.70%) | 0.301 |
| Other or Unknown Etiology (%) | 31 | 7 (22.60%) | 31 | 8 (25.80%) | 0.767 |
| **Endoscopic evaluation of EV (%)** | 31 | 19 (61.30%) | 31 | 21 (67.70%) | 0.596 |
| No EV (%) | 31 | 0 (0.00%) | 31 | 0 (0.00%) | NA |
| Mild EV (%) | 31 | 0 (0.00%) | 31 | 0 (0.00%) | NA |
| Moderate EV (%) | 31 | 2 (6.50%) | 31 | 2 (6.50%) | 1.000 |
| Severe EV (%) | 31 | 17 (54.80%) | 31 | 19 (61.30%) | 0.607 |
| **Laboratory Tests** |  |  |  |  |  |
| Red Blood Cell (10^12^/L) | 31 | 2.41 (0.79-4.68)  2.60±0.76 | 31 | 2.62 (1.30-4.22)  2.74±0.71 | 0.353 |
| Hemoglobin (g/L) | 31 | 71.00 (25.00-133.00)  72.96±22.71 | 31 | 72.00 (42.00-119.00)  75.13±19.51 | 0.513 |
| White Blood Cell (10^9^/L) | 31 | 5.30 (1.60-14.70)  5.61±3.10 | 31 | 4.50 (1.40-24.70)  5.93±4.61 | 0.866 |
| Platelet (10^9^/L) | 31 | 73.00 (23.00-222.00)  82.65±45.68 | 31 | 89.00 (17.00-263.00)  100.84±59.79 | 0.248 |
| Total Bilirubin (μmol/L) | 31 | 21.70 (8.80-184.00)  28.72±31.15 | 31 | 19.40 (9.10-87.20)  25.17±17.81 | 0.678 |
| Albumin (g/L) | 30 | 31.55 (14.30-45.60)  31.06±7.91 | 30 | 31.15 (13.60-42.80)  30.97±7.60 | 0.888 |
| Alanine Aminotransferase (U/L) | 31 | 24.00 (10.00-92.00)  31.19±21.94 | 31 | 25.00 (5.00-208.00)  39.23±39.39 | 0.526 |
| Aspartate Aminotransferase (U/L) | 31 | 29.00 (13.00-132.00)  44.52±34.10 | 31 | 31.00 (13.00-634.00)  59.32±109.42 | 0.833 |
| Alkaline Phosphatase (U/L) | 31 | 74.00 (40.00-444.00)  102.85±79.81 | 31 | 77.00 (38.70-391.00)  103.56±86.57 | 0.855 |
| Gamma-Glutamyl Transpeptidase (U/L) | 31 | 38.00 (9.00-544.00)  89.87±133.48 | 31 | 52.00 (12.00-708.00)  114.90±182.02 | 0.464 |
| Blood Urea Nitrogen (mmol/L) | 31 | 6.68 (4.07-28.25)  8.76±4.48 | 31 | 7.66 (2.22-15.16)  7.71±3.26 | 0.778 |
| Serum Creatinine (μmol/L) | 31 | 54.00 (29.00-169.00)  63.38±27.53 | 31 | 69.00 (38.00-211.00)  72.39±33.62 | 0.190 |
| Potassium (mmol/L) | 31 | 4.10 (3.03-5.39)  4.04±0.48 | 31 | 4.06 (3.28-5.00)  4.05±0.36 | 0.916 |
| Sodium (mmol/L) | 31 | 138.90 (130.40-145.40)  138.49±3.61 | 31 | 138.50 (130.50-146.20)  137.87±3.69 | 0.531 |
| Prothrombin Time (seconds) | 31 | 15.40 (11.00-20.90)  15.47±1.89 | 31 | 15.00 (11.30-36.60)  16.43±4.73 | 0.978 |
| INR | 31 | 1.23 (0.79-1.88)  1.24±0.20 | 31 | 1.18 (0.82-3.73)  1.35±0.55 | 0.916 |
| APTT (seconds) | 31 | 39.60 (28.70-59.20)  39.44±6.21 | 31 | 39.70 (33.00-66.90)  41.92±8.08 | 0.473 |
| **Child-Pugh Score** | 31 | 7.00 (5.00-10.00)  7.23±1.28 | 31 | 7.00 (5.00-13.00)  7.23±2.19 | 0.346 |
| **Child-Pugh Class A/B+C (%)** | 31 | 8 (25.80%)/23 (74.20%) | 31 | 13 (41.90%)/18 (58.10%) | 0.180 |
| **MELD Score** | 31 | 5.30 (-3.24-13.90)  5.56±4.33 | 31 | 5.12 (-1.19-27.43)  6.98±6.06 | 0.443 |
| **Endoscopic** **variceal treatment (%)** | 31 | 20 (64.50%) | 31 | 21 (67.70%) | 0.788 |
| **Vasoactive drugs (%)** | 31 | 31 (100.00%) | 31 | 31 (100.00%) | NA |
| Somatostatin (%) | 31 | 27 (87.10%) | 31 | 27 (87.10%) | 1.000 |
| Octreotide (%) | 31 | 23 (74.20%) | 31 | 19 (61.30%) | 0.277 |
| **Proton-pump inhibitor (%)** | 31 | 31 (100.00%) | 31 | 31 (100.00%) | NA |
| **Antibiotics (%)** | 31 | 19 (61.30%) | 31 | 20 (64.50%) | 0.793 |
| **Red blood cell transfusion (%)** | 31 | 21 (67.70%) | 31 | 18 (58.10%) | 0.430 |
| **5-day rebleeding (%)** | 31 | 6 (19.40%) | 31 | 3 (9.70%) | 0.279 |
| **In-hospital death (%)** | 31 | 3 (9.70%) | 31 | 1 (3.20%) | 0.301 |

**Abbreviations:** Pts: Patients; HBV: Hepatic B Virus; HCV: Hepatic C Virus; AUGIB: Acute Upper Gastrointestinal Bleeding; INR: International Standardization Ratio; APTT: Activated Partial Thromboplastin Time; MELD: Model for End-stage Liver Disease; EV: Esophageal Varices; NA: Not Available.

**Supplementary Table 6. PSM analysis of difference between Yunnan Baiyao and no hemostatic drug groups**

| **Variables** | **No. Pts** | **Yunnan Baiyao group** | **No. Pts** | **No hemostatic drug group** | **P value** |
| --- | --- | --- | --- | --- | --- |
| **Age (years)** | 49 | 55.39 (30.08-82.60)  56.01±11.28 | 49 | 53.36 (24.92-82.51)  55.67±11.85 | 0.741 |
| **Sex (male) (%)** | 49 | 34 (69.40%) | 49 | 34 (69.40%) | 1.000 |
| **Cancer (%)** | 49 | 9 (18.40%) | 49 | 8 (16.30%) | 0.790 |
| Liver cancer (%) | 49 | 8 (16.30%) | 49 | 7 (14.30%) | 0.779 |
| Extrahepatic cancer (%) | 49 | 1 (2.00%) | 49 | 1 (2.00%) | 1.000 |
| **Clinical features of AUGIB (%)** |  |  |  |  |  |
| Hematemesis (%) | 49 | 26 (53.10%) | 49 | 22 (44.90%) | 0.419 |
| Melena (%) | 49 | 42 (85.70%) | 49 | 43 (87.80%) | 0.766 |
| Both hematemesis and melena (%) | 49 | 19 (38.80%) | 49 | 16 (32.70%) | 0.527 |
| **Etiology of Liver Diseases** | 49 |  | 49 |  | 0.292 |
| HBV (%) | 49 | 22 (44.90%) | 49 | 17 (34.70%) | 0.302 |
| HCV (%) | 49 | 3 (6.10%) | 49 | 2 (4.10%) | 0.646 |
| Alcohol Abuse (%) | 49 | 13 (26.50%) | 49 | 19 (38.80%) | 0.196 |
| HBV+Alcohol Abuse (%) | 49 | 4 (8.20%) | 49 | 5 (10.20%) | 0.727 |
| HCV+Alcohol Abuse (%) | 49 | 0 (0.00%) | 49 | 0 (0.00%) | NA |
| Drug Related Liver Diseases (%) | 49 | 5 (10.20%) | 49 | 2 (4.10%) | 0.239 |
| Autoimmune Liver Diseases (%) | 49 | 2 (4.10%) | 49 | 3 (6.10%) | 0.646 |
| Other or Unknown Etiology (%) | 49 | 8 (16.30%) | 49 | 12 (24.50%) | 0.316 |
| **Endoscopic evaluation of EV (%)** | 49 | 28 (57.10%) | 49 | 34 (69.40%) | 0.209 |
| No EV (%) | 49 | 3 (6.10%) | 49 | 4 (8.20%) | 0.695 |
| Mild EV (%) | 49 | 3 (6.10%) | 49 | 0 (0.00%) | 0.079 |
| Moderate EV (%) | 49 | 1 (2.00%) | 49 | 4 (8.20%) | 0.168 |
| Severe EV (%) | 49 | 21 (42.90%) | 49 | 26 (53.10%) | 0.312 |
| **Laboratory Tests** |  |  |  |  |  |
| Red Blood Cell (10^12^/L) | 49 | 2.47 (0.98-4.33)  2.61±0.73 | 49 | 2.84 (1.21-4.22)  2.81±0.67 | 0.063 |
| Hemoglobin (g/L) | 49 | 70.00 (19.00-136.00)  74.29±25.76 | 49 | 75.00 (42.00-119.00)  75.86±19.05 | 0.513 |
| White Blood Cell (10^9^/L) | 49 | 5.50 (0.90-29.10)  6.18±4.73 | 49 | 4.50 (1.10-24.70)  5.50±4.09 | 0.451 |
| Platelet (10^9^/L) | 49 | 77.00 (21.00-458.00)  105.35±87.47 | 49 | 87.00 (17.00-293.00)  98.47±54.82 | 0.506 |
| Total Bilirubin (μmol/L) | 49 | 20.50 (5.30-184.00)  29.89±32.81 | 49 | 24.90 (7.40-105.30)  29.50±22.16 | 0.392 |
| Albumin (g/L) | 48 | 29.50 (14.00-47.20)  29.18±7.32 | 48 | 32.35 (13.60-48.00)  32.85±7.59 | ***0.015*** |
| Alanine Aminotransferase (U/L) | 49 | 29.50 (14.00-47.20)  29.18±7.32 | 49 | 23.00 (5.00-208.00)  34.61±35.30 | 0.208 |
| Aspartate Aminotransferase (U/L) | 49 | 29.00 (9.00-1399.00)  62.96±198.81 | 49 | 31.00 (13.00-634.00)  51.51±88.49 | 0.127 |
| Alkaline Phosphatase (U/L) | 49 | 74.00 (30.00-685.00)  98.89±102.05 | 49 | 79.00 (28.00-391.00)  105.18±76.97 | 0.212 |
| Gamma-Glutamyl Transpeptidase (U/L) | 49 | 30.00 (8.00-737.00)  68.00±126.82 | 49 | 51.00 (12.00-708.00)  118.22±171.29 | ***0.021*** |
| Blood Urea Nitrogen (mmol/L) | 49 | 6.84 (2.53-19.05)  7.76±3.73 | 49 | 6.12 (2.77-13.46)  6.87±2.91 | 0.279 |
| Serum Creatinine (μmol/L) | 49 | 56.00 (31.00-121.00)  59.72±17.33 | 49 | 54.30 (28.00-211.00)  62.04±30.03 | 0.672 |
| Potassium (mmol/L) | 49 | 4.09 (3.09-5.29)  4.12±0.43 | 49 | 4.00 (2.79-5.00)  3.98±0.37 | 0.147 |
| Sodium (mmol/L) | 49 | 138.20 (123.50-145.70)  137.15±4.70 | 49 | 137.60 (122.60-146.20)  137.18±4.60 | 0.963 |
| Prothrombin Time (seconds) | 49 | 15.80 (12.70-36.10)  16.74±3.82 | 49 | 14.90 (11.30-36.60)  16.13±4.18 | 0.113 |
| INR | 49 | 1.26 (0.94-3.57)  1.38±0.43 | 49 | 1.17 (0.82-3.73)  1.32±0.48 | 0.127 |
| APTT (seconds) | 49 | 40.90 (28.70-61.30)  40.34±6.56 | 49 | 40.50 (28.60-66.90)  42.12±7.87 | 0.416 |
| **Child-Pugh Score** | 49 | 7.00 (5.00-11.00)  7.61±1.75 | 49 | 7.00 (5.00-13.00)  7.10±2.07 | 0.081 |
| **Child-Pugh Class A/B/C (%)** | 49 | 13 (26.50%)25  (51.00%)/11 (22.40%) | 49 | 23 (46.90%)19  (38.80%)/7 (14.30%) | 0.106 |
| **MELD Score** | 49 | 6.61 (-1.75-16.33)  5.56±4.33 | 49 | 5.12 (-1.19-27.43)  6.98±6.06 | 0.462 |
| **Endoscopic** **variceal treatment (%)** | 49 | 27 (55.10%) | 49 | 31 (63.30%) | 0.411 |
| **Vasoactive drugs (%)** | 49 | 45 (91.80%) | 49 | 45 (91.80%) | 1.000 |
| Somatostatin (%) | 49 | 36 (73.50%) | 49 | 32 (65.30%) | 0.381 |
| Octreotide (%) | 49 | 26 (53.10%) | 49 | 31 (63.30%) | 0.306 |
| **Proton-pump inhibitor (%)** | 49 | 49 (100.00%) | 49 | 49 (100.00%) | NA |
| **Antibiotics (%)** | 49 | 28 (57.10%) | 49 | 26 (53.10%) | 0.685 |
| **Red blood cell transfusion (%)** | 49 | 29 (59.20%) | 49 | 28 (57.10%) | 0.838 |
| **5-day rebleeding (%)** | 49 | 13 (26.50%) | 49 | 2 (4.10%) | ***0.002*** |
| **In-hospital death (%)** | 49 | 6 (12.20%) | 49 | 2 (4.10%) | 0.140 |

**Abbreviations:** Pts: Patients; HBV: Hepatic B Virus; HCV: Hepatic C Virus; AUGIB: Acute Upper Gastrointestinal Bleeding; INR: International Standardization Ratio; APTT: Activated Partial Thromboplastin Time; MELD: Model for End-stage Liver Disease; NA: Not Available; EV: Esophageal Varices.

**Supplementary Table 7. PSM analysis of difference between norepinephrine and no hemostatic drug groups**

| **Variables** | **No. Pts** | **Norepinephrine group** | **No. Pts** | **No hemostatic drug group** | **P value** |
| --- | --- | --- | --- | --- | --- |
| **Age (years)** | 48 | 53.88 (31.05-84.56)  55.03±11.64 | 48 | 52.85 (29.85-76.49)  54.08±10.33 | 0.703 |
| **Sex (male) (%)** | 48 | 37 (77.10%) | 48 | 35 (72.90%) | 0.637 |
| **Cancer (%)** | 48 | 8 (16.70%) | 48 | 4 (8.30%) | 0.217 |
| Liver cancer (%) | 48 | 6 (12.50%) | 48 | 3 (6.20%) | 0.294 |
| Extrahepatic cancer (%) | 48 | 2 (4.20%) | 48 | 1 (2.10%) | 0.557 |
| **Clinical features of AUGIB (%)** |  |  |  |  |  |
| Hematemesis (%) | 48 | 23 (47.90%) | 48 | 24 (50.00%) | 0.838 |
| Melena (%) | 48 | 42 (87.50%) | 48 | 38 (79.20%) | 0.273 |
| Both hematemesis and melena (%) | 48 | 17 (35.40%) | 48 | 14 (29.20%) | 0.513 |
| **Etiology of Liver Diseases** | 48 |  | 48 |  | 0.248 |
| HBV (%) | 48 | 25 (52.10%) | 48 | 19 (39.60%) | 0.219 |
| HCV (%) | 48 | 4 (8.30%) | 48 | 2 (4.20%) | 0.399 |
| Alcohol Abuse (%) | 48 | 16 (33.30%) | 48 | 16 (33.30%) | 1.000 |
| HBV+Alcohol Abuse (%) | 48 | 8 (16.70%) | 48 | 6 (12.50%) | 0.563 |
| HCV+Alcohol Abuse (%) | 48 | 0 (0.00%) | 48 | 1 (2.10%) | 0.315 |
| Drug Related Liver Diseases (%) | 48 | 1 (2.10%) | 48 | 3 (6.20%) | 0.307 |
| Autoimmune Liver Diseases (%) | 48 | 0 (0.00%) | 48 | 5 (10.40%) | ***0.022*** |
| Other or Unknown Etiology (%) | 48 | 10 (20.80%) | 48 | 10 (20.80%) | 1.000 |
| **Endoscopic evaluation of EV (%)** | 48 | 36 (75.00%) | 48 | 32 (66.70%) | 0.369 |
| No EV (%) | 48 | 2 (4.20%) | 48 | 1 (2.10%) | 0.557 |
| Mild EV (%) | 48 | 1 (2.10%) | 48 | 0 (0.00%) | 0.315 |
| Moderate EV (%) | 48 | 5 (10.40%) | 48 | 4 (8.30%) | 0.726 |
| Severe EV (%) | 48 | 28 (58.30%) | 48 | 26 (56.20%) | 0.837 |
| **Laboratory Tests** |  |  |  |  |  |
| Red Blood Cell (10^12^/L) | 48 | 2.74 (1.39-4.25)  2.77±0.72 | 48 | 2.66 (1.30-4.22)  2.68±0.60 | 0.529 |
| Hemoglobin (g/L) | 48 | 77.50 (31.00-128.00)  78.94±24.01 | 48 | 72.00 (31.00-110.00)  71.56±16.93 | 0.129 |
| White Blood Cell (10^9^/L) | 48 | 3.85 (1.20-16.00)  4.60±2.86 | 48 | 4.20 (1.60-24.70)  6.12±5.85 | 0.446 |
| Platelet (10^9^/L) | 48 | 73.00 (17.00-222.00)  79.04±37.60 | 48 | 81.50 (17.00-333.00)  96.65±63.29 | 0.305 |
| Total Bilirubin (μmol/L) | 48 | 22.40 (8.40-90.40)  27.05±17.04 | 48 | 17.00 (5.90-197.60)  28.28±31.11 | 0.220 |
| Albumin (g/L) | 48 | 32.50 (10.00-39.90)  30.69±6.16 | 47 | 31.40 (13.60-48.00)  31.35±8.56 | 0.655 |
| Alanine Aminotransferase (U/L) | 48 | 27.50 (7.00-347.00)  46.00±60.26 | 48 | 24.50 (8.00-208.00)  45.31±50.20 | 0.869 |
| Aspartate Aminotransferase (U/L) | 48 | 34.50 (12.00-773.00)  71.33±130.78 | 48 | 31.00 (14.00-634.00)  83.02±149.24 | 0.750 |
| Alkaline Phosphatase (U/L) | 48 | 68.45 (41.00-388.00)  89.13±63.12 | 48 | 78.00 (38.70-440.00)  109.56±83.37 | 0.180 |
| Gamma-Glutamyl Transpeptidase (U/L) | 48 | 44.00 (6.00-683.00)  89.77±130.89 | 48 | 61.00 (10.00-708.00)  116.56±150.98 | 0.204 |
| Blood Urea Nitrogen (mmol/L) | 48 | 6.72 (2.03-19.26)  8.03±4.05 | 48 | 7.75 (2.22-19.82)  7.93±3.80 | 0.997 |
| Serum Creatinine (μmol/L) | 48 | 58.00 (32.00-119.00)  58.69±16.95 | 48 | 60.50 (28.00-274.00)  73.32±50.25 | 0.331 |
| Potassium (mmol/L) | 46 | 4.00 (3.00-5.50)  4.04±0.45 | 48 | 4.01 (3.28-5.00)  4.01±0.35 | 0.904 |
| Sodium (mmol/L) | 46 | 137.80 (130.40-151.40)  138.26±4.33 | 48 | 138.70 (122.60-160.80)  138.69±5.96 | 0.407 |
| Prothrombin Time (seconds) | 48 | 16.35 (12.90-49.50)  17.82±5.64 | 48 | 14.95 (10.80-36.60)  17.05±5.91 | ***0.028*** |
| INR | 48 | 1.33 (0.99-5.94)  1.51±0.74 | 48 | 1.18 (0.77-3.73)  1.43±0.69 | ***0.027*** |
| APTT (seconds) | 48 | 40.90 (27.30-67.10)  41.75±7.35 | 48 | 40.75 (28.40-66.90)  42.52±8.57 | 0.863 |
| **Child-Pugh Score** | 48 | 7.00 (5.00-13.00)  7.58±1.96 | 48 | 7.00 (5.00-13.00)  7.48±2.33 | 0.515 |
| **Child-Pugh Class A/B/C (%)** | 48 | 16 (33.30%)/24  (43.80%)/8 (16.70%) | 48 | 20 (41.70%)/18  (37.50%)/10 (20.80%) | 0.467 |
| **MELD Score** | 48 | 5.96 (-3.37-21.06)  6.91±5.30 | 48 | 4.70 (-7.52-27.43)  6.82±7.90 | 0.379 |
| **Endoscopic** **variceal treatment (%)** | 48 | 31 (64.60%) | 48 | 30 (62.50%) | 0.832 |
| **Vasoactive drug (%)** | 48 | 42 (87.50%) | 48 | 42 (87.50%) | 1.000 |
| Somatostatin (%) | 48 | 40 (83.30%) | 48 | 35 (72.90%) | 0.217 |
| Octreotide (%) | 48 | 12 (25.00%) | 48 | 28 (58.30%) | ***0.001*** |
| Proton-pump inhibitor (%) | 48 | 48 (100.00%) | 48 | 46 (95.80%) | 0.153 |
| **Antibiotics (%)** | 48 | 22 (45.80%) | 48 | 24 (50.00%) | 0.683 |
| **Red blood cell transfusion (%)** | 48 | 25 (52.10%) | 48 | 30 (62.50%) | 0.302 |
| **5-day rebleeding (%)** | 48 | 10 (20.80%) | 48 | 3 (6.20%) | ***0.037*** |
| **In-hospital death (%)** | 48 | 0 (0.00%) | 48 | 4 (8.30%) | ***0.041*** |

**Abbreviations:** Pts: Patients; HBV: Hepatic B Virus; HCV: Hepatic C Virus; AUGIB: Acute Upper Gastrointestinal Bleeding; INR: International Standardization Ratio; APTT: Activated Partial Thromboplastin Time; MELD: Model for End-stage Liver Disease; EV: Esophageal Varices.

**Supplementary Table 8. PSM analysis of difference between carbazochrome sodium sulfonate and no hemostatic drug groups**

| **Variables** | **No. Pts** | **Carbazochrome sodium sulfonate group** | **No. Pts** | **No hemostatic drug group** | **P value** |
| --- | --- | --- | --- | --- | --- |
| **Age (years)** | 31 | 55.65 (42.22-78.70)  56.79±8.08 | 31 | 60.08 (39.00-76.49)  59.60±10.97 | 0.307 |
| **Sex (male) (%)** | 31 | 21 (67.70%) | 31 | 19 (61.30%) | 0.596 |
| **Cancer (%)** | 31 | 4 (12.90%) | 31 | 4 (12.90%) | 1.000 |
| Liver cancer (%) | 31 | 3 (9.70%) | 31 | 3 (9.70%) | 1.000 |
| Extrahepatic cancer (%) | 31 | 1 (3.20%) | 31 | 1 (3.20%) | 1.000 |
| **Clinical features of AUGIB (%)** |  |  |  |  |  |
| Hematemesis (%) | 31 | 13 (41.90%) | 31 | 12 (38.70%) | 0.796 |
| Melena (%) | 31 | 27 (87.10%) | 31 | 26 (83.90%) | 0.718 |
| Both hematemesis and melena (%) | 31 | 9 (29.00%) | 31 | 7 (22.60%) | 0.562 |
| **Etiology of Liver Diseases** | 31 |  | 31 |  | 0.134 |
| HBV (%) | 31 | 10 (32.30%) | 31 | 8 (25.80%) | 0.576 |
| HCV (%) | 31 | 2 (6.50%) | 31 | 2 (6.50%) | 1.000 |
| Alcohol Abuse (%) | 31 | 11 (35.50%) | 31 | 12 (38.70%) | 0.793 |
| HBV+Alcohol Abuse (%) | 31 | 1 (3.20%) | 31 | 4 (12.90%) | 0.162 |
| HCV+Alcohol Abuse (%) | 31 | 1 (3.20%) | 31 | 0 (0.00%) | 0.313 |
| Drug Related Liver Diseases (%) | 31 | 0 (0.00%) | 31 | 5 (16.10%) | ***0.020*** |
| Autoimmune Liver Diseases (%) | 31 | 2 (6.50%) | 31 | 3 (9.70%) | 0.641 |
| Other or Unknown Etiology (%) | 31 | 8 (25.80%) | 31 | 5 (16.10%) | 0.349 |
| **Endoscopic evaluation of EV (%)** | 31 | 20 (64.50%) | 31 | 18 (58.10%) | 0.602 |
| No EV (%) | 31 | 2 (6.50%) | 31 | 2 (6.50%) | 1.000 |
| Mild EV (%) | 31 | 1 (3.20%) | 31 | 0 (0.00%) | 0.313 |
| Moderate EV (%) | 31 | 1 (3.20%) | 31 | 0 (0.00%) | 0.313 |
| Severe EV (%) | 31 | 16 (51.60%) | 31 | 16 (51.60%) | 1.000 |
| **Laboratory Tests** |  |  |  |  |  |
| Red Blood Cell (10^12^/L) | 31 | 2.64 (1.28-5.10)  2.79±0.82 | 31 | 2.96 (1.79-3.75)  2.90±0.52 | 0.278 |
| Hemoglobin (g/L) | 31 | 80.00 (36.00-150.00)  83.52±28.23 | 31 | 81.00 (42.00-119.00)  83.39±16.08 | 0.678 |
| White Blood Cell (10^9^/L) | 31 | 5.30 (1.40-17.70)  6.21±3.98 | 31 | 4.90 (1.60-13.10)  5.68±2.90 | 0.860 |
| Platelet (10^9^/L) | 31 | 73.00 (13.00-289.00)  95.58±63.88 | 31 | 97.00 (36.00-251.00)  108.16±56.10 | 0.275 |
| Total Bilirubin (μmol/L) | 31 | 19.60 (5.50-83.70)  27.66±20.72 | 31 | 26.40 (6.60-87.20)  29.37±21.48 | 0.673 |
| Albumin (g/L) | 31 | 29.80 (14.30-48.00)  30.71±7.79 | 30 | 30.45 (23.00-46.30)  31.67±6.59 | 0.609 |
| Alanine Aminotransferase (U/L) | 31 | 20.00 (6.00-265.00)  31.32±45.01 | 31 | 28.00 (14.00-110.00)  37.06±25.31 | ***0.015*** |
| Aspartate Aminotransferase (U/L) | 31 | 33.00 (12.00-426.00)  53.35±74.89 | 31 | 33.00 (12.00-426.00)  53.35±74.89 | 0.151 |
| Alkaline Phosphatase (U/L) | 31 | 78.00 (41.70-688.00)  123.33±155.06 | 31 | 84.00 (38.70-391.00)  125.93±95.45 | 0.426 |
| Gamma-Glutamyl Transpeptidase (U/L) | 31 | 49.00 (9.00-755.00)  152.94±224.87 | 31 | 62.00 (14.00-708.00)  160.13±209.25 | 0.573 |
| Blood Urea Nitrogen (mmol/L) | 31 | 7.08 (3.28-23.00)  8.41±4.41 | 31 | 6.32 (3.36-41.82)  7.98±6.81 | 0.324 |
| Serum Creatinine (μmol/L) | 31 | 52.00 (33.00-234.00)  67.56±45.89 | 31 | 61.00 (28.00-919.00)  88.16±155.47 | 0.607 |
| Potassium (mmol/L) | 31 | 3.94 (3.03-4.90)  3.99±0.51 | 30 | 4.10 (3.44-5.00)  4.07±0.32 | 0.466 |
| Sodium (mmol/L) | 31 | 138.90 (129.40-143.90)  138.32±3.37 | 30 | 138.05 (130.10-143.90)  138.02±3.91 | 0.735 |
| Prothrombin Time (seconds) | 31 | 15.50 (12.40-23.60)  16.09±2.34 | 31 | 14.80 (10.80-21.30)  15.18±2.80 | 0.076 |
| INR | 31 | 1.25 (0.92-2.10)  1.30±0.24 | 31 | 1.13 (0.77-1.95)  1.22±0.31 | 0.073 |
| APTT (seconds) | 31 | 41.50 (31.80-51.50)  40.58±5.47 | 31 | 39.40 (28.40-50.70)  39.41±5.61 | 0.468 |
| **Child-Pugh Score** | 31 | 7.00 (5.00-11.00)  7.71±1.70 | 31 | 7.00 (5.00-12.00)  7.45±2.35 | 0.339 |
| **Child-Pugh Class A/B/C (%)** | 31 | 7 (22.60%)/19  (61.30%)/5 (16.10%) | 31 | 14 (45.20%)/11  (35.50%)/6 (19.40%) | 0.102 |
| **MELD Score** | 31 | 5.82 (-5.22-20.33)  6.06±5.70 | 31 | 5.38 (-3.21-24.23)  5.83±6.26 | 0.795 |
| **Endoscopic** **variceal treatment (%)** | 31 | 16 (51.60%) | 31 | 15 (48.40%) | 0.799 |
| **Vasoactive drugs (%)** | 31 | 28 (90.30%) | 31 | 28 (90.30%) | 1.000 |
| Somatostatin (%) | 31 | 28 (90.30%) | 31 | 23 (74.20%) | 0.096 |
| Octreotide (%) | 31 | 26 (83.90%) | 31 | 17 (54.80%) | ***0.013*** |
| **Proton-pump inhibitor (%)** | 31 | 30 (96.80%) | 31 | 31 (100.00%) | 0.313 |
| **Antibiotics (%)** | 31 | 20 (64.50%) | 31 | 20 (64.50%) | 1.000 |
| **Red blood cell transfusion (%)** | 31 | 16 (51.60%) | 31 | 13 (41.90%) | 0.445 |
| **5-day rebleeding (%)** | 31 | 3 (9.70%) | 31 | 1 (3.20%) | 0.301 |
| **In-hospital death (%)** | 31 | 2 (6.50%) | 31 | 0 (0.00%) | 0.151 |

**Abbreviations:** Pts: Patients; HBV: Hepatic B Virus; HCV: Hepatic C Virus; AUGIB: Acute Upper Gastrointestinal Bleeding; INR: International Standardization Ratio; APTT: Activated Partial Thromboplastin Time; MELD: Model for End-stage Liver Disease; EV: Esophageal Varices.

**Supplementary Table 9. PSM analysis of difference between vitamin K and no hemostatic drug groups**

| **Variables** | **No. Pts** | **Vitamin K group** | **No. Pts** | **No hemostatic drug group** | **P value** |
| --- | --- | --- | --- | --- | --- |
| **Age (years)** | 32 | 54.18 (23.41-80.06)  55.90±11.82 | 32 | 55.78 (29.85-84.77)  57.75±13.16 | 0.717 |
| **Sex (male) (%)** | 32 | 25 (78.10%) | 32 | 25 (78.10%) | 1.000 |
| **Cancer (%)** | 32 | 7 (21.90%) | 32 | 5 (15.60%) | 0.522 |
| Liver cancer (%) | 32 | 6 (18.80%) | 32 | 5 (15.60%) | 0.740 |
| Extrahepatic cancer (%) | 32 | 1 (3.10%) | 32 | 0 (0.00%) | 0.313 |
| **Clinical features of AUGIB (%)** |  |  |  |  |  |
| Hematemesis (%) | 32 | 17 (53.10%) | 32 | 17 (53.10%) | 1.000 |
| Melena (%) | 32 | 24 (75.00%) | 32 | 27 (84.40%) | 0.351 |
| Both hematemesis and melena (%) | 32 | 9 (28.10%) | 32 | 12 (37.50%) | 0.424 |
| **Etiology of Liver Diseases** | 32 |  | 32 |  | 0.170 |
| HBV (%) | 32 | 17 (53.10%) | 32 | 11 (34.40%) | 0.131 |
| HCV (%) | 32 | 2 (6.20%) | 32 | 3 (9.40%) | 0.641 |
| Alcohol Abuse (%) | 32 | 12 (37.50%) | 32 | 10 (31.20%) | 0.599 |
| HBV+Alcohol Abuse (%) | 32 | 3 (9.40%) | 32 | 3 (9.40%) | 1.000 |
| HCV+Alcohol Abuse (%) | 32 | 0 (0.00%) | 32 | 1 (3.10%) | 0.313 |
| Drug Related Liver Diseases (%) | 32 | 0 (0.00%) | 32 | 1 (3.10%) | 0.313 |
| Autoimmune Liver Diseases (%) | 32 | 0 (0.00%) | 32 | 4 (12.50%) | ***0.039*** |
| Other or Unknown Etiology (%) | 32 | 4 (12.50%) | 32 | 8 (25.00%) | 0.200 |
| **Endoscopic evaluation of EV (%)** | 32 | 13 (40.60%) | 32 | 19 (59.40%) | 0.134 |
| No EV (%) | 32 | 1 (3.10%) | 32 | 2 (6.20%) | 0.554 |
| Mild EV (%) | 32 | 1 (3.10%) | 32 | 1 (3.10%) | 1.000 |
| Moderate EV (%) | 32 | 1 (3.10%) | 32 | 2 (6.20%) | 0.554 |
| Severe EV (%) | 32 | 10 (31.20%) | 32 | 14 (43.80%) | 0.302 |
| **Laboratory Tests** |  |  |  |  |  |
| Red Blood Cell (10^12^/L) | 32 | 2.70 (1.65-3.62)  2.67±0.56 | 32 | 2.66 (1.63-3.96)  2.73±0.63 | 0.778 |
| Hemoglobin (g/L) | 32 | 77.00 (48.00-127.00)  79.78±22.61 | 32 | 75.00 (42.00-119.00)  80.00±20.79 | 0.872 |
| White Blood Cell (10^9^/L) | 32 | 5.45 (0.90-23.60)  5.77±4.15 | 32 | 5.75 (1.10-30.70)  6.53±5.39 | 0.573 |
| Platelet (10^9^/L) | 32 | 65.00 (9.00-369.00)  88.75±68.38 | 32 | 82.00 (32.00-842.00)  121.53±140.88 | 0.151 |
| Total Bilirubin (μmol/L) | 32 | 28.70 (9.20-271.70)  56.28±69.78 | 32 | 28.85 (5.90-241.40)  37.40±42.21 | 0.638 |
| Albumin (g/L) | 32 | 31.30 (17.20-41.80)  30.04±6.50 | 31 | 29.50 (21.40-43.50)  30.53±5.98 | 0.929 |
| Alanine Aminotransferase (U/L) | 32 | 27.50 (9.00-344.00)  53.06±73.05 | 32 | 27.00 (5.00-438.00)  48.97±75.55 | 0.946 |
| Aspartate Aminotransferase (U/L) | 32 | 34.50 (11.00-1104.00)  102.50±203.10 | 32 | 41.50 (13.00-994.00)  76.25±170.06 | 0.835 |
| Alkaline Phosphatase (U/L) | 32 | 70.00 (34.00-337.00)  95.17±67.40 | 32 | 85.50 (42.90-450.00)  119.48±94.00 | 0.138 |
| Gamma-Glutamyl Transpeptidase (U/L) | 32 | 41.50 (7.00-423.00)  78.69±87.02 | 32 | 52.50 (10.00-994.00)  143.84±221.46 | 0.298 |
| Blood Urea Nitrogen (mmol/L) | 32 | 8.66 (2.28-20.94)  8.62±4.10 | 32 | 6.26 (2.22-55.01)  10.33±10.88 | 0.624 |
| Serum Creatinine (μmol/L) | 32 | 55.00 (36.00-449.00)  72.13±71.54 | 32 | 67.00 (28.00-919.00)  97.26±154.01 | 0.214 |
| Potassium (mmol/L) | 32 | 4.10 (3.26-5.27)  4.14±0.47 | 32 | 4.02 (2.98-5.80)  4.09±0.55 | 0.481 |
| Sodium (mmol/L) | 32 | 137.75 (130.00-146.00)  138.24±3.67 | 32 | 137.15 (130.10-146.50)  137.32±4.30 | 0.379 |
| Prothrombin Time (seconds) | 32 | 16.45 (12.70-34.20)  17.61±3.90 | 32 | 15.55 (12.20-40.90)  16.93±5.25 | 0.106 |
| INR | 32 | 1.37 (0.98-3.24)  1.47±0.48 | 32 | 1.22 (0.89-4.19)  1.41±0.60 | 0.121 |
| APTT (seconds) | 32 | 42.55 (30.30-75.40)  43.91±8.63 | 32 | 43.55 (30.20-57.70)  42.90±6.76 | 0.957 |
| **Child-Pugh Score** | 32 | 8.00 (5.00-13.00)  8.13±2.06 | 32 | 7.00 (5.00-14.00)  7.78±2.38 | 0.323 |
| **Child-Pugh Class A/B/C (%)** | 32 | 7 (21.90%)/17  (53.10%)/8 (25.00%) | 32 | 10 (31.20%)16  (50.00%)/6 (18.80%) | 0.655 |
| **MELD Score** | 32 | 7.82 (1.02-32.06)  9.16±7.08 | 32 | 6.71 (0.91-40.95)  8.84±7.65 | 0.819 |
| **Endoscopic** **variceal treatment (%)** | 32 | 11 (34.40%) | 32 | 14 (43.80%) | 0.442 |
| **Vasoactive drugs (%)** | 32 | 28 (87.50%) | 32 | 28 (87.50%) | 1.000 |
| Somatostatin (%) | 32 | 24 (75.00%) | 32 | 22 (68.80%) | 0.578 |
| Octreotide (%) | 32 | 19 (59.40%) | 32 | 17 (53.10%) | 0.614 |
| **Proton-pump inhibitor (%)** | 32 | 31 (96.90%) | 32 | 31 (96.90%) | 1.000 |
| **Antibiotics (%)** | 32 | 21 (65.60%) | 32 | 20 (62.50%) | 0.794 |
| **Red blood cell transfusion (%)** | 32 | 17 (53.10%) | 32 | 18(56.20%) | 0.802 |
| **5-day rebleeding (%)** | 32 | 10 (31.20%) | 32 | 2 (6.20%) | ***0.010*** |
| **In-hospital death (%)** | 32 | 5 (15.60%) | 32 | 1 (3.10%) | 0.086 |

**Abbreviations:** Pts: Patients; HBV: Hepatic B Virus; HCV: Hepatic C Virus; AUGIB: Acute Upper Gastrointestinal Bleeding; INR: International Standardization Ratio; APTT: Activated Partial Thromboplastin Time; MELD: Model for End-stage Liver Disease; EV: Esophageal Varices.
